# Challenges in Maintaining the Hemostatic Balance in Children Undergoing Extracorporeal Membrane Oxygenation: A Systematic Literature Review

**DOI:** 10.3389/fped.2020.612467

**Published:** 2020-12-16

**Authors:** Joppe G. F. Drop, Enno D. Wildschut, Sabrina T. G. Gunput, Matthijs de Hoog, C. Heleen van Ommen

**Affiliations:** ^1^Department of Pediatric Hematology, Erasmus MC Sophia Children's Hospital, Rotterdam, Netherlands; ^2^Department of Intensive Care and Pediatric Surgery, Erasmus MC Sophia Children's Hospital, Rotterdam, Netherlands; ^3^Department of Medical Library, Erasmus MC, University Medical Center, Rotterdam, Netherlands

**Keywords:** pediatrics, risk factor, bleeding, thrombosis, bivalirudin, unfractionated heparin (UFH), extra corporeal membrane oxygenation (ECMO)

## Abstract

**Background:** Despite advances in technology and clinical experience, the incidence of hemostatic complications, including bleeding and thrombosis, remains high in children supported with extracorporeal membrane oxygenation (ECMO). These hemostatic complications are important to prevent, since they are associated with increased morbidity and mortality. This systematic literature review aims to outline the most important risk factors for hemostatic complications in children undergoing ECMO treatment, to summarize the reported alternative anticoagulant drugs used in pediatric ECMO and to describe studied associations between coagulation tests and hemostatic complications.

**Methods:** A literature search was performed in Embase, Medline, Web of Science Core Collection, Cochrane Central Register of Controlled Trials, and Google Scholar in February 2020. Included studies were studies evaluating children (<18 years old) treated with ECMO, and studies evaluating risk factors for hemostatic complications, alternative anticoagulants, or the association between coagulation tests and hemostatic complications.

**Results:** Out of 1,152 articles, 35 studies were included. Thirteen out of 49 risk factors were investigated in three or more studies. Most consistent results were found regarding ECMO duration and pH. However, evidence for risk factors was equivocal in the majority of studies, which is explained by the variability of populations studied, definitions of hemostatic complications, ECMO circuits, anticoagulation protocols, transfusion triggers and monitoring of anticoagulation. Five studies described alternative anticoagulants, including bivalirudin (*n* = 3), argatroban (*n* = 1) and FUT (*n* = 1). Higher anti-factor Xa levels were associated with less clotting events in one of nine studies, investigating the association between tests and hemostatic complications. Two studies revealed an association between anti-factor Xa assay-based protocols and a decreased number of transfusions, bleedings and need for circuit change.

**Conclusion:** Studies regarding risk factors showed conflicting results and a few retrospective studies reported the use of new anticoagulants and data on coagulation tests in relation to hemostatic complications. To decrease hemostatic complications in ECMO children, prospective multicenter studies are needed with clear bleeding and thrombotic definitions, and the best possible standardization of ECMO circuits used, anticoagulation protocols, and transfusion triggers.

## What This Study Adds

The incidence of hemostatic complications, including bleeding and thrombosis, in children undergoing extracorporeal membrane oxygenation (ECMO) is high. It is important to prevent hemostatic complications since these complications are associated with decreased survival. Unfractionated heparin is most frequently used to remain patency of the circuit and to prevent patient thrombosis. However, alternative anticoagulants including direct thrombin inhibitors have been described. To evaluate the hemostatic balance, several coagulation tests are used. Knowledge about risk groups for hemostatic complications, alternative anticoagulants, and the association between coagulation tests and bleeding and thrombotic complications in ECMO children may help decrease the number of hemostatic complications and improve outcome. Hence, this systematic review aims to assemble existing evidence of (1) risk factors for hemostatic complications, (2) suitable alternative anticoagulants, and (3) associations between coagulation tests and hemostatic complications in children undergoing ECMO support. Studies regarding risk factors showed conflicting results and a few retrospective studies reported the use of new anticoagulants and data on coagulation tests in relation to hemostatic complications. To decrease hemostatic complications in ECMO children, prospective multicenter studies are needed with clear bleeding and thrombotic definitions and the best possible standardization of ECMO circuits used, anticoagulation protocols, and transfusion triggers.

## Introduction

Extra Corporeal Membrane Oxygenation (ECMO) is the last treatment option for children with refractory cardiac and/or pulmonary failure. The methodology was introduced by Bartlett when he performed the first successful ECMO treatment in an infant in 1976 (1). Over time, through improvements in materials, components and techniques, the role of ECMO as a supportive therapy has expanded in pediatric patients (2). In 2019, the registry of the extracorporeal life support organization (ELSO) contained 12,850 ECMO runs from 430 participating centers worldwide (2). Neonatal and pediatric ECMO runs accounted for 33.5 and 21.6% of the total number of ECMO runs, respectively. Despite improvements in technology and increasing clinical expertise, hemostatic complications, including bleeding and thrombosis, remain an important cause of mortality and morbidity in ECMO treated children worldwide. Hemorrhagic complications, including intracranial hemorrhage, were reported in up to 29.1% of neonatal and 28.5% of pediatric ECMO patients. Thrombotic complications, including circuit thrombosis and cerebral infarction, arose in up to 16.7% of neonatal and 12.4% of pediatric patients (3). These hemostatic complications may be life threatening, especially when they occur in the central nervous system. In a multicenter, prospective study in 514 children with ECMO support, bleeding appeared to be independently associated with a higher risk of mortality (4). One of the policies to improve outcome in pediatric ECMO patients should, therefore, focus on decreasing the frequency of bleeding and thrombotic complications.

The hemostatic complications in children undergoing ECMO support are related to the continuous contact between circulating blood and the foreign surface of the extracorporeal circuit, shifting the hemostatic balance to a hypercoagulable state with risk of thrombosis. To restore the hemostatic balance and prevent thrombosis, the use of systemic anticoagulation is necessary, but also increases the risk of bleeding. Many other factors may be associated with the development of hemostatic complications, including circuit factors, such as mode of cannulation (venoarterial [VA] or venovenous [VV]), high shear stress causing acquired von Willebrand disease, and patient factors, such as developmental hemostasis, sepsis, and cardiopulmonary resuscitation (CPR) (5). Knowledge of important risk factors for hemostatic complications may help to define high risk groups for which the anticoagulation protocol can be adjusted. The type of anticoagulant drug may also have an effect on the frequency of hemostatic complications. Most centers use unfractionated heparin (UFH) owing to the short half-life and effective reversal ability by protamine sulfate (6, 7). Besides UFH, alternative anticoagulants like direct thrombin inhibitors (DTIs), including bilivarudin or argatroban, are increasingly used (8, 9). These DTIs get their name from their direct binding to thrombin to exert anticoagulant effects. They may induce a more stable anticoagulant effect leading to less hemostatic complications. In addition, the choice of coagulation test may affect the risk of hemostatic complications. To monitor the UFH effect, several coagulation tests are used, including activated clotting time (ACT), activated partial thromboplastin time (APTT), anti-Xa assays (aXa), thromboelastography (TEG), rotational thromboelastometry (ROTEM), or a combination of them in a variability of coagulation protocols (10). The best coagulation test in ECMO patients would be one which correlates well with the occurrence of hemostatic complications.

Knowledge about risk factors for hemostatic complications, the efficacy and safety of alternative anticoagulants, and the association between coagulation tests and bleeding and thrombotic complications in ECMO children may help decrease the number of hemostatic complications and improve outcome. Hence, this systematic review aims to assemble existing evidence of (1) risk factors for hemostatic complications, (2) suitable alternative anticoagulants, and (3) correlations between coagulation tests and hemostatic complications in children undergoing ECMO support.

## Methods

This systematic review was performed in agreement with the Preferred Reporting Items for Systematic Reviews and Meta-Analyses (PRISMA) guidelines (11). This review is registered in the PROSPERO database (CRD42019133803). Embase.com, Medline ALL via Ovid, Cochrane Central Register of Controlled Trials, Web of Science Core Collection and Google scholar (top 100) were systematically searched from inception until February 27th, 2020. The search strategy is attached in Supplementary Material 1.

### Study Selection

In this systematic review the following studies were included: all studies evaluating pediatric patients (<18 years old) treated with ECMO, and studies evaluating risk factors for hemostatic complications, alternative anticoagulants, or the association between coagulation tests and hemostatic complications in children treated with ECMO. Outcome was defined as any hemostatic complication and comprised of all hemorrhagic and thrombotic complications. Furthermore, only English language and human studies were included. Case-reports, editorials, conference abstracts and letters, and unavailable full-text articles were excluded.

Two independent review authors (JD, HO) screened titles and abstracts to select eligible studies. Disagreements about study selection were resolved by discussion. One reviewer (JD) examined the full text records to determine which studies met the in- and exclusion criteria. Any doubt was resolved via discussion and consensus with the second author (HO).

After study selection based on the in- and exclusion criteria, the Newcastle Ottawa Score (NOS) was used to assess the methodological quality of studies describing risk factors (12). This scale assesses patient selection bias, comparability of cases and controls and determination of outcome. The NOS values are depicted in Supplementary Material 2. Studies with a NOS above five were considered high quality and were included in this manuscript.

### Data Extraction

Extracted data from eligible studies investigating risk factors for hemostatic complications included number of patients, authors, year of publication, study design, years of patient inclusion, outcome, odds ratios (ORs) or mean values and corresponding *P*-values. A list of all risk factors for hemostatic complications was drafted and risk factors examined in three or more studies were included in this manuscript. Outcome measures were OR or relative risk (RR) estimates with corresponding 95% confidence intervals (CIs). If a risk factor was described in multiple types of analyses (i.e., multivariate, univariate, comparing means or incidence) for the same outcome, only the highest level of evidence was considered eligible for this review. Continuous variables were appraised higher than categorical variables. Extracted data from eligible studies investigating alternative anticoagulants involved type of anticoagulant, used dosages and target ranges, reason for use, patient characteristics, and bleeding and thrombotic complications. Obtained data from studies examining the association between coagulation tests and hemostatic complications included type of tests and hemostatic complications, patient characteristics and the associations found.

## Results

### Study Identification

**Figure 1:**
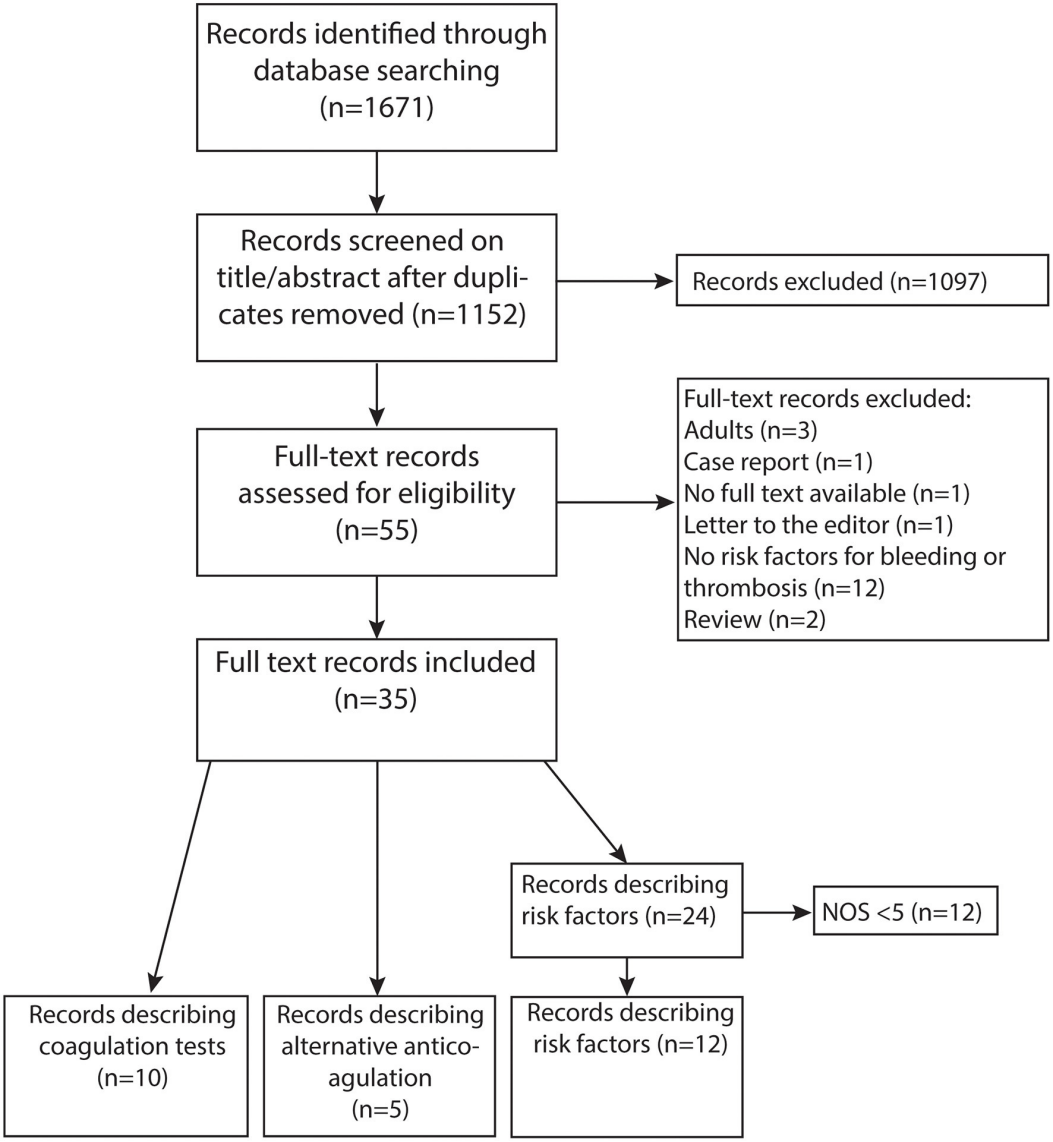
PRISMA flow diagram of literature search (11).

The systematic search strategy yielded 1,152 articles (Figure 1). The majority of studies was excluded (*n* = 1,097), as they did not fulfill the eligibility criteria after screening of titles and abstracts. A total of 35 out of 55 studies were included after full text screening: 20 studies investigated risk factors for hemostatic complications, five studies described alternative anticoagulants, six studies examined the association between coagulation tests and hemostatic complications, and four studies described both risk factors and the association between coagulation tests and hemostatic complications. Twelve studies were considered low quality and excluded. A substantial part of studies had a retrospective design. The years of publication ranged from 1986 to 2020 and 2013 was the median year of publication. Eleven studies retrospectively investigated the ELSO registry.

### Risk Factors for Hemostatic Complications

Qualitative assessment was performed in 24 studies concerning risk factors for hemostatic complications. After application of NOS, twelve studies were considered high quality, including 11 retrospective studies and one prospective observational study. In these 12 studies, a total of 49 potential risk factors was described. (Supplementary Material 3) 13 risk factors were investigated in three or more studies and included in this manuscript. Eight studies performed multivariate analyses, which are described in Tables 1, 2. The average NOS score of included studies was 5.66.

**Table 1:** Risk factors for bleeding complications in pediatric ECMO studies with multivariate analyses.

**Author**	**Year**	**Study population**	**Study type**	**Outcome**	**Risk factors**	**Univariate OR**	***p***	**Multivariate OR**	***p***
Hardart and Fackler (13)	1999	3,777 neonates	Retrospective ELSO review (1992–1995)	ICH	Age Gestational age <34 weeks 34– <36 weeks 36– <38 weeks CPR/cardiac arrest pre-ECMO Gender (male) PaCO_2_ >50 mmHg Birthweight (kg) <2.5 2.5– <3.0 Coagulopathy/DIC Primary diagnosis sepsis pH <7.0 7.0– <7.2 7.2– <7.35 Mode: VV ECMO vs. VA ECMO	0.71 11.6 5.0 2.5 1.9 1.3 1.8 3.3 1.9 2.5 2.6 3.9 2.6 1.5 0.7 (0.53-0.92)	<0.001–0.01 <0.001 <0.001 <0.001 <0.001 0.01–0.05 <0.001 <0.001 <0.001 <0.001 <0.001 <0.001 <0.001 <0.001 <0.05	ns 12.1 (6.6–22.0) 4.1 (2.9–5.8) 2.1 (1.6–2.8) ns ns ns ns ns 1.6 (1.1–2.2) 1.8 (1.4–2.4) 2.5 (1.6–3.9) 1.8 (1.3–2.5) 1.6 (1.1–2.2) 0.97 (0.73–1.30)	0.0001 0.0001 0.0001 0.0009 0.0001 0.0001 0.0001 0.0001
Hardartet al. (14)	2004	1,398 neonates <37 weeks gestation	Retrospective ELSO review (1992–2000)	ICH	Age Gestational age CPR/cardiac arrest pre-ECMO Gender (male) Increasing PaCO_2_ Birthweight ECMO mode Primary diagnosis sepsis pH <7.0 7.0– <7.2		0.01 0.09 <0.001 ns 0.03 ns ns	ns – ns – ns – – 1.78 (1.24–2.56) 2.41 (1.27–4.56) 1.65 (1.10–2.45)	0.004 0.0004
De Molet al. (15)	2008	25 newborns with ICH vs. 40 control patients matched for diagnosis, gestational age, birth weight	Retrospective case control study	ICH	Age Gestational age CPR pre-ECMO PH ≤ 7.3 6 h pre- ECMO PaCO_2_ ≥ 45 mmHg 6 h pre-ECMO ECMO duration Birth weight	1.0 (0.9–1.0) 0.4 (0.1–1.4) 2.0 (0.4–9.0) 4.5 (1.4–14.0) 3.3 (1.0–10.9) ns ns		– – – ns ns	
Churchet al. (16)	2017	752 neonates between 29 and 34 weeks of gestation	Retrospective ELSO review (1976–2008)	ICH	Gestational age ECMO mode Birthweight CPR/cardiac arrest pre-ECMO	ns ns		0.59 (0.37–0.93) 1.42 (0.83–2.43)	0.02 0.20
Daltonet al. (4)	2017	514 children <19 years	Prospective cohort study	Daily bleeding event	Neonatal age CPR/cardiac arrest pre-ECMO ECMO indication Cardiac vs. Resp. CPR vs. Resp.ECMO duration			1.04 (1.02–1.05) 1.76 (1.45–2.13) 1.34 (1.11–1.63) 1.52 (1.16–1.98) Significant	<0.001 <0.001 0.002

*ECMO, extracorporeal membrane oxygenation; ELSO, extracorporeal life support organization; CPR, cardiopulmonary resuscitation; ns, not significant; ICH, intracranial hemorrhage; VV, venovenous; VA, venoarterial; OR, odds ratio; DIC, disseminated intravascular coagulation*.

**Table 2:** Risk factors for thrombotic events in studies with multivariate analyses.

**Author**	**Year**	**Study population**	**Study type**	**Outcome**	**Risk factors**	**Univariate OR (95%CI)**	***p***	**Multivariate OR (95%CI)**	***p***
Church et al. (16)	2017	752 neonates between 29 and 34 weeks of gestation	Retrospective ELSO review (1976–2008)	Cerebral infarct	Birthweight ECMO mode Gestational age DIC		ns ns	0.79 (0.64–0.98) 3.01 (1.39–1.75)	0.03 0.04
Rollins et al. (17)	2012	2,617 children 1 mo- <18 years respiratory failure	Retrospective ELSO review (1993–2007)	CNS infarct or hemorrhage	Age Male gender Weight (kg) Cardiac arrest pre ECMO paCO_2_ pre-ECMO DIC Hours on ECMO pH pre ECMO pH 6.4–7.18 pH 7.19–7.29 pH >7.29 ref group ECMO mode (VA vs. VV)		ns <0.05 <0.05 <0.05 <0.05 <0.05 <0.05 <0.05 <0.05	0.96 (0.93–0.98) – ns ns ns ns ns 2.1 (1.5–2.8) 1.5 (1.1–2.1) ref group 1.6 (1.1–2.3)	
Werho et al. (18)	2015	3,517 cardiac surgical patients ECMO postop. <18 years	Retrospective ELSO review (2002–2013)	Stroke	Neonatal age Weight for age z score Duration of ECMO ≥ 167 h Cannulation site: neck	1.73 (1.41–2.14) 1.06 (0.98–1.15) 1.45 (1.16–1.80) 1.38 (1.03–1.83)	<0.0001 0.13 0.001 0.03	1.77 (1.32–2.36) 1.14 (1.04–1.25) 1.38 (1.06–1.78) 1.29 (0.91–1.81)	0.0001 0.004 0.02 0.15
Dalton et al. (4)	2017	514 children <19 years	Prospective cohort study	Daily thrombotic event	ECMO mode (VV vs. VA) ECMO duration			0.62 (0.40–0.95) Significant	0.024
Maul et al. (19)	2020	274 patients with 299 ECMO runs	Retrospective cohort study	Circuit change	Weight (mean) ECMO duration (mean) ECMO mode (VV vs. VA) Respiratory indication		ns <0.05 <0.1 <0.05	– 1.007 (1.004–1.010) – 2.8 (1.4–5.8)	<0.05 <0.05

*ECMO, extracorporeal membrane oxygenation; ELSO, extracorporeal life support organization; CPR, cardiopulmonary resuscitation; ns, not significant; VV, venovenous; VA, venoarterial; OR, odds ratio; DIC, disseminated intravascular coagulation; h, hour; postop, postoperatively; kg, kilogram; CNS, central nervous system*.

#### Age at ECMO Initiation

Age at ECMO initiation as risk factor for hemostatic complications was investigated in eight studies (4, 13–15, 17, 18, 20, 21). Dalton and colleagues performed a prospective, multicenter cohort study, investigating 514 children undergoing ECMO support. It was concluded that neonatal age was associated with a significantly increased risk of daily bleeding events (RR 1.04, 95%CI 1.02–1.05, *p* < 0.01), defined as an intracranial hemorrhage or blood loss that required transfusion (4). In addition, in the ELSO study of Rollins et al. examining 2617 ECMO patients between 1 month and 18 years old with respiratory failure, increasing age was associated with decreased odds of CNS infarction or hemorrhage (17). However, no significant association could be found in two retrospective ELSO reviews of Hardart et al. and in the case-control study by Mol et al. (13–15) Contradictory results were also found for thrombotic complications. In the retrospective study of Muensterer et al., age at cannulation was 83 ± 108 h in 112 patients without clotted circuits and 70 ± 111 h in 62 patients with clotted circuits (*p* = 0.45) (20). In addition, no association was found between age and change of circuit secondary to thrombus formation in 62 pediatric patients in the retrospective study of Irby et al. (21). However, Werho et al. concluded that neonatal age was associated with significantly increased odds of stroke from a retrospective ELSO study with 3,517 cardiac surgical patients requiring ECMO postoperatively (18).

#### Gestational Age

Gestational age was investigated in four studies (13–16). Increasing gestational age between 29 and 34 weeks was found to be significantly associated with decreasing odds of cerebral infarction (OR 0.79, 95%CI 0.64–0.98, *p* = 0.03), but not with ICH in a retrospective ELSO study of 752 neonates by Church et al. (16). In both the study of de Mol et al., a retrospective case control study, and in the retrospective ELSO review of Hardart in 2004, gestational age was also not significantly associated with ICH (14, 15). However, in 1999 a positive association was found by Hardart et al.: newborns with a gestational age below 34 weeks (OR 12.1, 95%CI 6.6–22.0, *p* = 0.0001), 34–36 weeks (OR 4.1, 95%CI 2.9–5,8, *p* = 0.0001) and 36–38 weeks (OR 2.1, 95%CI 1.6–2.8, *p* = 0.0001) had a significantly increased risk of ICH (13).

#### CPR/Cardiac Arrest Pre-ECMO

Four studies investigated the association between CPR and/or pre-ECMO cardiac arrest and hemostatic complications (13, 15–17). In three retrospective ELSO studies in various patient groups, no association could be found in the multivariate analyses (13, 16, 17). In addition, the incidence of children with CPR before ECMO did not significantly differ between cases and controls in the study of de Mol et al. (15).

#### Gender

In five studies, gender was not correlated with a hemostatic complication in a univariate analysis (14, 15, 17, 20, 21). In the retrospective ELSO review of Hardart in 1999, 240 of 2,210 male patients (10.1%) had ICH (RR 1.3, *p* < 0.05). However, after adjustment for other significant predictors for ICH by multivariate analysis, no correlation was found (13).

#### PaCO_2_

Three studies examined the association between ICH and higher pre-ECMO PaCO_2_ in ECMO children (13, 15, 17). In the ELSO study of Rollins et al. the mean PaCO_2_ before ECMO was significantly higher in 233 children with CNS injury (64 mmHg; IQR 87 mmHg) than in 2,384 children without CNS injury (55 mmHg, IQR 75 mmHg, *p* < 0.05). However, after multivariate analysis no correlation was present (17). Similarly, no significant association was found in the multivariate analyses of another ELSO review of Hardart et al. in 2004 and in the case control study of de Mol et al. (14, 15).

#### (Birth)Weight

Eight studies investigated the association between bleeding or thrombotic events and (birth)weight, showing conflicting results (13–20). In the ELSO review of Church et al. lower birthweight increased the odds of ICH, but not for infarction in neonates between 29 and 34 weeks of gestation (OR 0.59, 95% CI 0.37–0.93, *p* = 0.02) (16). Three other studies with neonates did not find an association between birthweight and ICH after multivariate analyses (13–15). However, average cannulation weight of 62 neonates requiring a circuit change (2,988 g ± 602) was significantly lower than 112 neonates without a circuit change (3,212 g ± 580; *p* = 0.02), as concluded from the study of Muensterer (20).

In older children, lower weight-for-age z score was independently associated with stroke in cardiac surgical patients requiring ECMO postoperatively (OR 1.14, 95%CI 1.04–1.25, *p* = 0.004) (18). However, weight was not a risk factor for CNS injury in ECMO children with respiratory failure (17, 19). Furthermore, weight was not significantly different in children with circuit change (11 ± 14 kg) compared to children without circuit change (14 ± 21 kg, *p* > 0.05) (19).

#### Coagulopathy/Disseminated Intravascular Coagulation

The association between hemostatic complications and coagulopathy has been investigated in three studies (13, 16, 17). In the ELSO review of Hardart et al. in 1999, coagulopathy increased the odds for ICH in neonates significantly (OR 1.6, 95%CI 1.1–2.2, *p* = 0.0009) (13). In addition, the presence of DIC was associated with cerebral infarction in neonates between 29 and 34 weeks (OR 3.01, 95%CI 1.39–1.75, *p* = 0.04) in the ELSO review of Church et al. (16). In older children with ECMO due to respiratory failure, no association between DIC and CNS infarct or hemorrhage was found by Rollins et al. (17).

#### Platelet Count

Three studies have investigated platelet count and hemostatic complications (21–23). In a retrospective review of 25 children undergoing ECMO support by Sell et al., the incidence of ICH was significantly lower in patients with platelet count >100.000/uL and significantly higher in patients with platelet count <100.000/uL (23). Stallion et al. showed that in 42 children, the incidence of bleeding complications in patients managed with a platelet count >200.000/uL was not significantly different compared to patients with a goal platelet count >100.000/uL (22). In addition, platelet count was similar in 62 pediatric patients with or without circuit change due to thrombus formation in the retrospective study of Irby et al. (21).

#### Duration of ECMO Support

The association between duration of ECMO support and hemostatic complications has been described in six studies (4, 15, 17–20). In 2017, Dalton reported a strong association of ECMO duration in neonates and older children with the development of bleeding and thrombotic complications during the total ECMO period (4). In the study of Werho et al., ECMO duration >167 h increased the odds of stroke in cardiac surgical patients requiring ECMO postoperatively (OR 1.38, 95%CI 1.06–1.78, *p* = 0.02) (18). In addition, in the single center study of Maul et al., the risk for a circuit change increased with longer ECMO duration (OR 1.007, 95%CI 1.004–1.010, *p* < 0.05) (19). Moreover, Muensterer et al. reported an increased ECMO duration in patients with circuit clots (195 ± 108 h in patients without clots vs. 504 ± 224 h in patients with clotted circuits, *p* = 0.02) (20). In addition, in the ELSO review of Rollins et al., ECMO duration significantly increased the risk for CNS injury in children with respiratory failure (CNS injury 176 IQR 326, no CNS injury 201 IQR 356, *p* < 0.05) (17). However, ECMO duration was not associated with ICH in the study of de Mol et al.: ECMO duration was 155 ± 76 h in neonates with ICH and 171 ± 81 h (*p* = 0.43) in control patients (15).

#### ECMO Indication

ECMO indication is investigated in three studies (4, 19, 21). Both cardiac (OR 1.34, 95%CI 1.11–1.63, *p* < 0.002) and ECPR (OR 1.52, 95%CI 1.16–1.98, *p* < 0.002) indications in the study of Dalton et al. increased the risk for daily bleeding events compared to children with a respiratory ECMO indication (4). In contrast, in the study of Maul et al. the risk for circuit clots was increased in patients with respiratory indications (OR 2.8, 95%CI 1.4–5.8, *p* < 0.05) (19). However, no association between ECMO indications and circuit change was revealed in 62 pediatric patients from the single center of Irby et al. (*p* = 0.327) (21).

#### Mode of Cannulation

Mode of cannulation in relation to hemostatic complications was described in five studies (4, 13, 17–19) In the 2,617 children requiring ECMO support for respiratory failure, VA ECMO increased the odds of CNS infarction or hemorrhage compared to VV ECMO (OR 1.6, 95%CI 1.1–2.3) (17). Moreover, VV ECMO decreased the odds for daily thrombotic events, including patient and circuit related thrombosis, in the study of Dalton et al. (4). Maul et al., however, showed a trend toward a higher number of VV ECMO patients requiring circuit changes (19). In neonates, VV ECMO was not associated with significantly increased odds of ICH and cerebral infarction in three retrospective ELSO studies (13, 17, 18). In 3,517 cardiac surgical patients requiring ECMO postoperatively, cannulation in the neck did not increase the risk of hemostatic complications (18).

#### Sepsis as Primary Diagnosis

Sepsis as primary diagnosis has been described in four studies (13, 14, 16, 20). In the two retrospective ELSO studies of Hardert et al. comprising 3,777 and 1,398 neonates, sepsis as primary diagnosis increased the odds for ICH significantly (13, 14). In the retrospective review of Church et al., none of the primary diagnoses, including sepsis, were associated with ICH in neonates between 29 and 34 weeks of gestational age (16). In the study of Muensterer et al. sepsis as primary diagnosis was significantly less present in patients with clotted circuits (3%) than without clotted circuits (31%, *p* < 0.01) (20).

#### pH

Four studies described the association between pH and hemostatic complications (13–16). Additionally, the case control study of de Mol et al. showed an increased risk for ICH in neonates with pH <7.3 in children (15). In three ELSO registry studies, low pre-ECMO pH was associated with increased risk for ICH in neonates and for CNS infarction and hemorrhage in children (13, 14, 16).

### Summary of Risk Factors

Thirteen risk factors were described in at least three studies in various patient groups on ECMO. In the majority of these 13 risk factors, contradictory results were found. Most constant results were found between hemostatic complications and ECMO duration and pH: longer ECMO duration was associated with increased risk of bleeding and thrombotic complications in all age categories, and a low pre-ECMO pH was associated with an increased risk of intracranial injury in neonates.

### Alternative Anticoagulants

Five studies reported the use of alternative anticoagulants in pediatric ECMO patients (8, 9, 24–26). Three studies described bivalirudin, one case series covered the use of argatroban, and one case series outlined FUT. Nagle et al. reported the use of bivalirudin in pediatric patients on ECMO in a case series of 12 children with a median age of 8 days. All patients were managed with heparin initially, but anticoagulation was changed to bivalirudin due to heparin induced thrombocytopenia, heparin resistance, thrombus formation or unstable ACTs. The initial infusion ranged from 0.05 to 0.3 mg/kg/h. The maintenance dose that corresponded with an initial target APTT ranged from 0.045–0.48 mg/kg/h with a median rate of 0.16 mg/kg/h. Two patients suffered from bleeding from chest tubes requiring re-exploration and 8 patients had a circuit change, while on bivalirudin (9). Two groups of 21 post cardiotomy ECMO patients, including four neonates and six children, using UFH or bivalirudin were retrospectively compared in the study of Ranucci et al. Bivalirudin infusion was started at an initial dose of 0.03 to 0.05 mg/kg/h depending on the bleeding rate and adjusted according to ACT (target range 160–180 s), APTT (50–80 s) and r time values (12–30 min) of TEG. Blood loss and transfusion with platelets and fresh frozen plasma was significantly higher in the heparin group. The number of thrombotic events and mortality did not differ (25). Teruya et al. described six pediatric patients with bivalirudin on ECMO. The initial infusion rate was 0.05–0.15 mg/kg/h and was thereafter adjusted to maintain APTTs within a specific target range. The target range of 60–80 s was adjusted by the treating physician based on the patient condition (26).

Another direct thrombin inhibitor, argatroban, was described by Potter et al. in a case series of three children on ECMO. The circuit was primed with 30–50 ug argatroban on the basis of patient's ACT. Initial infusion ranged from 0.5 to 1 ug/kg/min and was titrated to individually set ACT target ranges between 250 and 300 s. None of the patients suffered from any significant hemorrhagic complications. However, all patients suffered from thromboembolic disease in varying severity during argatroban therapy (8).

FUT is a serine protease inhibitor with anticoagulant activity due to the inhibition of the coagulation and fibrinolytic systems (factor II, Xa, and XIIa). Due to its short half-life of 8 min it has been used in continuous renal replacement therapy (27, 28). Nagaya et al. presented evidence for the clinical use of FUT in 12 neonatal ECMO patients in whom hemorrhagic complications had occurred. They attempted to decrease only the patient's ACT levels, while keeping the ACT levels in the ECMO circuit at normal high levels. After administration of FUT in the drainage route, the heparin dose was decreased. FUT and heparin dosages were regulated to maintain the ACT at the reinfusion route at 190–220 s. The average dose of heparin was 21 ± 7.5 IU/kg/h and FUT 0.48 ± 0.22 mg/kg/h. In eight cases, the bleeding could be controlled by FUT administration. No difference was described in thrombotic formation in ECMO circuits between patients managed with FUT and heparin or heparin alone (24).

### Summary of Alternative Anticoagulants

The use of bivalirudin, argatroban and FUT in pediatric ECMO patients has been described in a total of 55 children, but pharmacokinetic data, clear dosing and monitoring guidelines are lacking.

### Association Between Coagulation Tests and Hemostatic Complications

Nine studies investigated the association between coagulation tests or coagulation protocol and hemostatic complications (21, 29–36). In an autopsy study of 29 pediatric ECMO non-survivors (median age 2 months), thrombosis and bleeding were diagnosed in 69 and 52% of the patients, respectively. ACT, APTT, and PT results of the last ECMO support day were not significantly associated with these hemostatic events (ACT: no hemorrhage group 214, hemorrhage group 209, *p* = 0.005, data APTT and PT not shown) (34). Additionally, ACT, APTT, and aXa levels did not show any differences during 24 and 72 h before a cerebrovascular event between 36 cases and controls in the study of Anton-Martin (29). Furthermore, in the study of O'Meara mean aXa levels, APTT and INR were not statistically different between the failed oxygenator (*n* = 7, PTT 137 ± 27;INR 1.5 ± 0.2) and no failed oxygenator group (*n* = 15, PTT 137 ± 45; INR 1.7 ± 0.5) (33). In the retrospective chart review of Grayck et al., the minimum and maximum ACT values did not significantly differ between 82 neonates with or without ICH (minimum: ICH 13 ± 7, no ICH 157 ± 4, *p* = 0.65; maximum ICH 271 ± 10, no ICH 254 ± 4, *p* = 0.90) (37). Irby et al. studied 62 mostly neonates and young infants with ECMO therapy of whom 17 patients required change of circuit or membrane oxygenator due to thrombosis. No difference in mean daily ACT measurements between patients with and without circuit or membrane oxygenator change was found (no circuit change: 195.26 ± 5.74; circuit change: 202.62 ± 3.96, *p* = 0.192). However, the mean aXa factor was significantly higher in the patients without thrombus formation (21). In the retrospective study of McMichael et al. of 69 pediatric ECMO patients, aXa levels and APTT were not significantly associated with bleeding or thrombotic complications (any bleed aXa 0.44 95%CI 0.23–0.44, no bleed aXa 0.43 95%CI 0.36–0.48, *p* = 0.56; any bleed APTT 79 95%CI 63–100, no bleed APTT 78 95%CI 67–97, *p* = 0.96; circuit clot formation aXa 0.44 95%CI 0.4–0.5, no circuit clot formation aXa 0.42 95%CI 0.36–0.47, *p* = 0.41; circuit clot formation APTT 78 95%CI 64–91, no circuit clot formation APTT 83 95%CI 67–101, *p* = 0.50) (30). Saini et al. investigated the value of a viscoelastic test, TEG-platelet mapping, in the prediction of bleeding complications in 24 children on ECMO. No difference in kaolin-activated heparinase TEG parameters were found between the bleeding and the non-bleeding group. Prediction of bleeding based on ROC revealed that the AUC for ADP-mediated platelet aggregation, AA-mediated platelet aggregation and ACT was 0.64 (95% CI: 0.48–0.79, *p* = 0.11), 0.68 (95%CI 0.53–0.83, *p* = 0.03), and 0.56 (95%CI 0.39–0.75, *p* = 0.42), respectively (36).

Northrop et al. showed that after initiation of a revised anticoagulation protocol, which included the incorporation of aXa, TEG and antithrombin measurements in addition to the standard laboratory tests ACT, PT/INR, and APTT, the median blood product usage, and the frequency of cannula and surgical site bleedings decreased. Moreover, the median circuit life increased from 3.6 to 4.3 days (*p* = 0.02) (32). Niebler et al. also showed a statistically significant association between a change from an ACT based anticoagulation protocol to an aXa based protocol and a decreased incidence of ICH and need for circuit change (31). Yu et al. prospectively compared a simple anticoagulation protocol of one center (ACT every 2 h and daily APTT and aXa levels) with an intensive anticoagulation protocol (ACT every 2 h, APTT, PT, and aXa every 12 h, daily antithrombin) in another center. No significant differences in outcomes including major bleeding [5 [15%] vs. 14 [23], *p* = 0.4], patient thrombosis (6 [18%] vs. 10 [16%], *p* = 0.8) were found, but the center with the extensive monitoring protocol performed significantly more circuit changes (19 [31%] vs. 3 [9%], *p* = 0.02) (35).

### Summary of Coagulation Tests

No clear association has been described between coagulation tests, such as APTT, AXA, ACT, INR, and TEG, and bleeding or thrombotic complications in pediatric ECMO patients. However, in one study higher anti-factor Xa levels were associated with less clotting events (21). Two studies revealed an association between anti-factor Xa assay-based protocols and a decreased number of transfusions, bleedings and need for circuit change (31, 32).

## Discussion

Hemostatic complications remain an important cause of morbidity and mortality during ECMO support in children (38). Over the last 6 years, the frequency of bleeding complications and circuit clotting has not changed significantly (39). Decreasing the number of hemostatic complications will improve outcome of pediatric ECMO patients. Unfortunately, this systematic literature review revealed conflicting results regarding most risk factors for hemostatic complications in pediatric ECMO patients and only a few studies reported the use of new methods of anticoagulation. In addition, data on coagulation tests in relation to hemostatic complications were rare.

This literature review shows that about 50 risk factors for hemostatic complications have been investigated in various neonatal and pediatric patient groups with ECMO support. The large number of risk factors studied reflects the multifactorial etiology and the complex and dynamic mechanisms of bleeding and thrombosis in ECMO patients. Some of these risk factors may contribute through similar pathways to a disrupted hemostatic balance, for example sepsis and DIC. In addition, the severity of the patient's condition changes during ECMO support contributing to an alternating risk of bleeding and thrombotic complications. The majority of papers had a retrospective design, resulting in an unclear detection and timing of hemostatic complications. However, performing prospective studies in ECMO patients is challenging due to difficulties with obtaining informed consent and gathering enough patients to provide sufficient statistical power. As result of the retrospective design, timing of thrombotic or bleeding events may have been unclear or these events may have been missed because they were not described in the patient file. Identifying risk factors for ICH has predominantly been performed in neonates with ECMO support. In the most recent ELSO registry report, 11% of neonates with ECMO support for respiratory or cardiac indications developed an ICH (40). In the first ELSO review, gestational age was significantly associated with ICH (13). However, in the subsequent ELSO reports, gestational age did not increase risk for ICH (14–16). This might be the result of improved technology over time. Sepsis as primary diagnosis was the most consistent risk factor for ICH in neonates. However, this risk factor was investigated in two ELSO registries with overlapping patient populations (13, 14). Duration of ECMO support and the last pH before ECMO initiation in neonates were consistent risk factors in this review. However, it is difficult to draw conclusions about the other potential risk factors as mostly contradictory results were found. This is partly explained by the various populations studied, and differences in definitions of bleeding and thrombotic complications among studies. In addition, ECMO circuits, anticoagulation protocols, transfusion triggers and monitoring of anticoagulation varied among centers participating in multicenter studies influencing the risk for bleeding and thrombotic complications. One clear definition of bleeding and thrombotic complications, which can be used in all future studies, is needed. Moreover, description of the circuit variables, anticoagulation and monitoring practices, and transfusion triggers in the publications may be helpful in comparing studies and interpret the risk for hemostatic complications.

The incidence of hemostatic complications may decrease by using an alternative anticoagulant drug for UFH. UFH is still the anticoagulant of choice in pediatric ECMO patients, due to its availability, reversibility by protamine, familiarity among physicians, and rapid onset of action. The use of anticoagulants other than UFH is rarely investigated in children undergoing ECMO support. This literature review revealed five studies, four of them concerning DTIs. DTIs have advantages over UFH, including direct binding of both circulating and clot-bound thrombin, resulting in increased efficacy compared to UFH, antithrombin independence and no risk of heparin-induced thrombocytopenia (41). Bivalirudin is the most commonly used alternative for UFH in pediatric patients. However, the dose range of bivalirudin as described in the available reports is rather large and no direct antidote exists. Furthermore, none of the easily available tests, such as ACT and APTT, are validated for DTI monitoring. The relationship between APTT and DTI concentration is non-linear, especially with high dosages of DTIs (42). Dedicated assays, based on a dilated thrombin time, are not available in all centers. Moreover, as concluded by this literature review, few data are available about the safety and efficacy of DTIs in pediatric ECMO patients. Large prospective, observational or controlled clinical trials with uniform thrombotic and bleeding definitions are needed to compare UFH with a DTI.

Optimal monitoring of anticoagulation may decrease the incidence of hemostatic complications as well. Unfortunately, the best monitoring strategy is still unknown. Several reports have investigated the relationship between ACT, APTT and aXa factor and heparin dose on ECMO (43). These data show that the correlation between aXa levels and heparin dose is better than that of APTT or ACT. Only few studies investigated the association between coagulation tests and hemostatic complications. Only one of them showed in 62 neonates that the mean aXa factor was significantly higher in neonates without thrombus formation. All studies were performed retrospectively, which may have hampered the identification of hemostatic complications, particularly the exact timing of them. Interestingly, two studies revealed an association between protocols incorporating aXa measurements and a decreased number of transfusions, bleedings and the need for a circuit change (31, 32). Since aXa has a good correlation with heparin dose, it is the most valuable test to monitor heparin dosing in neonatal and pediatric ECMO patients (39). However, aXa targets are variable between studies and aXa fails to monitor hypercoagulability or other derailments of the coagulation such as DIC. A multi test approach to monitor overall coagulation as well as an effective test for the anticoagulant administered seems necessary in managing pediatric ECMO patients. Future prospective studies investigating the association between hemostatic complications and separate or combinations of coagulation tests, including viscoelastic tests, will help to find the optimal strategy for anticoagulation monitoring in pediatric ECMO patients.

## Conclusion

To improve outcome of pediatric ECMO patients, a reduction of the frequency of hemostatic complications is required. To effectuate such a reduction, we should be aware of the risk factors for hemostatic complications, the safety and efficacy of alternative anticoagulants and the association between coagulation tests and hemostatic complications to improve monitoring of anticoagulant therapy. This review showed a lack of prospective studies, uniform definitions of outcome parameters and therefore inconsistent and conflicting data on risk factors, coagulation monitoring and alternative anticoagulant drugs. The large variation in studied risk factors, patient groups, used ECMO circuits, anticoagulation protocols, monitoring methods and transfusion triggers hinder further development in this field. To appropriately study risk factors, new anticoagulants and coagulation monitoring, prospective multicenter trials are needed with clear bleeding and thrombotic definitions, and the best possible standardization of abovementioned variables. This should be a joint effort of all disciplines who take care of pediatric patients on ECMO support.

## Data Availability Statement

The original contributions generated for this study are included in the article/Supplementary Material, further inquiries can be directed to the corresponding author/s.

## Author Contributions

JD and SG: performance of search. JD and CO: selection of studies. JD: drafting of tables. JD, EW, MdH, and CO: writing of manuscript. All authors: contributed to the article and approved the submitted version.

## Conflict of Interest

The authors declare that the research was conducted in the absence of any commercial or financial relationships that could be construed as a potential conflict of interest.

## Supplementary Material

Supplementary Material 1 Search strategy.

Supplementary Material 2 Table with all risk factors for bleeding or thrombosis in pediatric ECMO patients.

Supplementary Material 3 Newcastle Ottawa Scores.
