# Supplementary material for: Challenges in Maintaining the Hemostatic Balance in Children Undergoing Extracorporeal Membrane Oxygenation: A Systematic Literature Review
Source: Front Pediatr. 2020 Dec 16;8:612467. doi: 10.3389/fped.2020.612467 (PMC7772234; doi:10.3389/fped.2020.612467)
Supplement: Supplementary Material 1 — Search strategy. [file Table_1.docx]

**Search strategy**

**Used on 27-02-2020**

**Embase.com**

('bleeding'/exp OR 'thrombosis'/exp OR 'blood clotting'/exp OR 'anticoagulation'/de OR (Bleed* OR Hemorrhag* OR haemorrhag* OR Blood-clot* OR clotting OR Thromb* OR anticoagul* OR coagula*):ab,ti,kw) AND ('incidence'/exp OR 'thromboelastograph'/de OR 'thromboelastography'/de OR 'thromboelastometry'/de OR 'bleeding time'/exp OR 'blood clotting time'/de OR 'partial thromboplastin time'/exp OR 'prothrombin time'/de OR 'thrombin time'/de OR 'thromboplastin time'/de OR (incidence* OR Thromboelastograph* OR Thromboelastomet* OR ROTEM* OR TEG OR ((bleeding* OR clotting* OR coagulation* OR prothrombin* OR thrombin* OR thromboplastin*) NEXT/3 (time)) OR international-normali*-ratio OR INR OR ACT OR APTT OR PT OR PTT):ab,ti,kw) AND ('extracorporeal oxygenation'/de OR 'extracorporeal circulation'/de OR 'extracorporeal membrane oxygenation device'/de OR 'membrane oxygenator'/de OR 'mechanical circulatory support'/de OR (ECMO OR ECLS OR ((Extracorporeal* OR membrane* OR sheet*) NEAR/6 (oxygen* OR life-support* OR circulat* OR treat* OR therap*)) OR mechanic*-circul*-support*):ab,ti,kw) AND (child/exp OR adolescent/exp OR adolescence/exp OR 'child behavior'/de OR 'child parent relation'/de OR pediatrics/exp OR childhood/exp OR 'child nutrition'/de OR 'infant nutrition'/exp OR 'child welfare'/de OR 'child abuse'/de OR 'child advocacy'/de OR 'child development'/de OR 'child growth'/de OR 'child health'/de OR 'child health care'/exp OR 'child care'/exp OR 'childhood disease'/exp OR 'child death'/de OR 'child psychiatry'/de OR 'child psychology'/de OR 'pediatric ward'/de OR 'pediatric hospital'/de OR 'pediatric anesthesia'/de OR 'pediatric intensive care unit'/de OR 'neonatal intensive care unit'/de OR (adolescen* OR preadolescen* OR infan* OR newborn* OR (new NEXT/1 born*) OR baby OR babies OR neonat* OR child* OR kid OR kids OR toddler* OR teen* OR boy* OR girl* OR minors OR underag* OR (under NEXT/1 (age* OR aging)) OR juvenil* OR youth* OR kindergar* OR puber* OR pubescen* OR prepubescen* OR prepubert* OR pediatric* OR paediatric* OR school* OR preschool* OR highschool* OR suckling* OR PICU OR NICU OR PICUs OR NICUs):ab,ti,kw)

**Medline (Ovid)**

(exp Hemorrhage/ OR exp Thrombosis/ OR exp Blood Coagulation/ OR (Bleed* OR Hemorrhag* OR haemorrhag* OR Blood-clot* OR clotting OR Thromb* OR anticoagul* OR coagula*).ab,ti,kf.) AND (Incidence/ OR thromboelastograph/ OR thromboelastography/ OR thromboelastometry/ OR Bleeding Time/ OR Whole Blood Coagulation Time/ OR Partial Thromboplastin Time/ OR Prothrombin Time/ OR Thrombin Time/ OR (incidence* OR Thromboelastograph* OR Thromboelastomet* OR ROTEM* OR TEG OR ((bleeding* OR clotting* OR coagulation* OR prothrombin* OR thrombin* OR thromboplastin*) ADJ3 (time)) OR international-normali*-ratio OR INR OR ACT OR APTT OR PT OR PTT).ab,ti,kf.) AND (extracorporeal therapy/ OR extracorporeal membrane oxygenation device/ OR membrane oxygenator/ OR (ECMO OR ECLS OR ((Extracorporeal* OR membrane* OR sheet*) ADJ6 (oxygen* OR life-support* OR circulat* OR treat* OR therap*)) OR mechanic*-circul*-support*).ab,ti,kf.) AND (exp Child/ OR exp Infant/ OR exp Adolescent/ OR exp "Child Behavior"/ OR exp "Parent Child Relations"/ OR exp "Pediatrics"/ OR "Child Nutrition Sciences"/ OR "Infant nutritional physiological phenomena"/ OR exp "Child Welfare"/ OR "Child Development"/ OR exp "Child Health Services"/ OR exp "Child Care"/ OR "Child Rearing"/ OR exp "Child development Disorders, Pervasive"/ OR "Child Psychiatry"/ OR "Child Psychology"/ OR "Hospitals, Pediatric"/ OR exp "Intensive Care Units, Pediatric"/ OR (adolescen* OR infan* OR newborn* OR (new ADJ born*) OR baby OR babies OR neonat* OR child* OR kid OR kids OR toddler* OR teen* OR boy* OR girl* OR minors OR underag* OR (under ADJ1 (age* OR aging)) OR juvenil* OR youth* OR kindergar* OR puber* OR pubescen* OR prepubescen* OR prepubert* OR pediatric* OR paediatric* OR school* OR preschool* OR highschool* OR suckling* OR PICU OR NICU OR PICUs OR NICUs).ab,ti,kf.)

**Web of Science**

TS=(((Bleed* OR Hemorrhag* OR haemorrhag* OR Blood-clot* OR clotting OR Thromb* OR anticoagul* OR coagula*)) AND ((incidence* OR Thromboelastograph* OR Thromboelastomet* OR ROTEM* OR TEG OR ((bleeding* OR clotting* OR coagulation* OR prothrombin* OR thrombin* OR thromboplastin*) NEAR/2 (time)) OR international-normali*-ratio OR INR OR ACT OR APTT OR PT OR PTT)) AND ((ECMO OR ECLS OR ((Extracorporeal* OR membrane* OR sheet*) NEAR/5 (oxygen* OR life-support* OR circulat* OR treat* OR therap*)) OR mechanic*-circul*-support*)) AND ((adolescen* OR preadolescen* OR infan* OR newborn* OR (new NEAR/1 born*) OR baby OR babies OR neonat* OR child* OR kid OR kids OR toddler* OR teen* OR boy* OR girl* OR minors OR underag* OR (under NEAR/1 (age* OR aging)) OR juvenil* OR youth* OR kindergar* OR puber* OR pubescen* OR prepubescen* OR prepubert* OR pediatric* OR paediatric* OR school* OR preschool* OR highschool* OR suckling* OR PICU OR NICU OR PICUs OR NICUs)))

**Cochrane CENTRAL**

((Bleed* OR Hemorrhag* OR haemorrhag* OR (Blood NEXT/1 clot*) OR clotting OR Thromb* OR anticoagul* OR coagula*):ab,ti) AND ((incidence* OR Thromboelastograph* OR Thromboelastomet* OR ROTEM* OR TEG OR ((bleeding* OR clotting* OR coagulation* OR prothrombin* OR thrombin* OR thromboplastin*) NEXT/3 (time)) OR (international NEXT/1 normali* NEXT/1 ratio) OR INR OR ACT OR APTT OR PT OR PTT):ab,ti) AND ((ECMO OR ECLS OR ((Extracorporeal* OR membrane* OR sheet*) NEAR/6 (oxygen* OR "life-support*" OR circulat* OR treat* OR therap*)) OR (mechanic* NEXT/1 circul* NEXT/1 support*)):ab,ti) AND ((adolescen* OR preadolescen* OR infan* OR newborn* OR (new NEXT/1 born*) OR baby OR babies OR neonat* OR child* OR kid OR kids OR toddler* OR teen* OR boy* OR girl* OR minors OR underag* OR (under NEXT/1 (age* OR aging)) OR juvenil* OR youth* OR kindergar* OR puber* OR pubescen* OR prepubescen* OR prepubert* OR pediatric* OR paediatric* OR school* OR preschool* OR highschool* OR suckling* OR PICU OR NICU OR PICUs OR NICUs):ab,ti)

**Google Scholar**  *Top 100 relevant references*

bleeding|hemorrhage|haemorrhage|clotting|thrombus|thrombosis|coagulation|incidence|thromboelastograph|ROTEM|TEG ECMO|ECLS|"extracorporeal|extra-corporeal oxygenation" child|children|baby|babies|kid|kids|boy|girl|boys|girls|pediatric|paediatric|PICU|NICU|PICUs|NICUs
