# Supplementary material for: Challenges in Maintaining the Hemostatic Balance in Children Undergoing Extracorporeal Membrane Oxygenation: A Systematic Literature Review
Source: Front Pediatr. 2020 Dec 16;8:612467. doi: 10.3389/fped.2020.612467 (PMC7772234; doi:10.3389/fped.2020.612467)
Supplement: Supplementary Material 2 — Table with all risk factors for bleeding or thrombosis in pediatric ECMO patients. [file Table_2.pdf]

Supplemental material 2 Table with all risk factors for bleeding or thrombosis in pediatric ECMO patients.

Demographic risk factors associated with bleeding or thrombosis in pediatric ECMO patients.

ACA aminocaproic acid, ARDS acute respiratory distress syndrome, BP blood pressure, BPM beats per minute, CC circuit change, CDH congenital diaphragmatic hernia, CI confidence interval, CM cardiomyopathy, CPB cardiopulmonary bypass, CPR cardiopulmonary resuscitation, DIC diffuse intravascular coagulation, dTGA transposition of great arteries, E excessive bleeding, ECMO extracorporeal membrane oxygenation, ECPR extracorporeal cardiopulmonary resuscitation, ELSO extracorporeal life support organization, FFP fresh frozen plasma, ICH intracranial hemorrhage, IQR interquartile range, kg kilogram, MAP mean arterial pressure, NC no circuit change, NE non excessive, OR odds ratio, PC platelet count, PEEP positive end-expiratory pressure, PIP peak inspiratory pressure, pRBC packed red blood cells, RDS Respiratory distress syndrome, RR relative risk, TAPVR total anomalous pulmonary venous return, VA venoarterial, VV venovenous.

**Risk factor**	**Patient population (n)**	**Author**	**Study**	**Means/ Univariate/ Multivariate**	**Outcome/ event**	**Results**	**P-value**
Airway pressure (mean)<br>>30<br>20-30	104 children with ICH from ELSO	Hardart, 1999	Retrospective ELSO review between 1992-1995	Univariate	ICH	RR 2.4<br>RR 1.4	<0.05<br>NS
Age (years)	514 children	Dalton, 2017	Retrospective analysis	Multivariate	Daily bleeding events	RR 1.04 (1.02-1.05)	<0.01
Age (years)	62 in total, 45 without circuit change, 17 with circuit change	Irby, 2014	Retrospective singe institution study.	Means	Circuit change	No circuit change: 3.2 (SD 5.6)<br>One circuit change: 0.2 (SD 0.4)<br>Multiple circuit changes: 0.2 (SD 0.3)	0.132
Age (days)	1898 children with congenital heart disease	Polito, 2015	Retrospective ELSO review between 2005-2010	Means	Stroke/ICH	Stroke/ICH: 7 (1, 19)<br>No stroke/ICH: 7 (1, 20)	0.06
Age (months)	36 cases (ICH & stroke) vs 36	Anton-Martin, 2017	Retrospective single center individually	Means	ICH & stroke	Cases: 4.8 (range 0–158.4)<br>Controls: 7.9 (range 0–202.8)	0.81

	controls (no ICH/stroke)		matched case-control study				
Age (months)	24 patients on whom TEG was performed during ECMO support between 9/1/2011 to 12/31/2012	Saini, 2016	Retrospective single-center chart review	Means	Severe bleeding	No severe bleeding: 9 (0.8–12)<br>Severe bleeding: 48 (2.7–90)	0.40
Age (days)	2617 children with respiratory failure	Rollins, 2012	Retrospective ELSO between 1993 and 2007	Means	CNS injury	Yes: 366 (IQR 81- 1093)<br>No: 415 (IQR 106- 2188)	<0.05
Age (years)	2617 children with respiratory failure	Rollins, 2012	Retrospective ELSO between 1993 and 2007	Multivariate	CNS injury	OR 0.96 (95% CI 0.93-0.98)	Not mentioned
Age at cannulation (h)	164 children	Muensterer, 2011	Retrospective singe center study	Means	Circuit change	No circuit change: 83 (SD 108)<br>Circuit change: 70 (SD 111)	0.45
Neonate at ECMO	62 in total, 45 without circuit change, 17 with circuit change	Irby, 2014	Retrospective singe institution study.	Means	Circuit change	No circuit change: neonate 24 (53%), no neonate 21 (47%)<br>One circuit change: neonate 4 (33%), no neonate (67%)<br>Multiple circuit change: neonate 2 (40), no neonate 3 (60)	0.434
Neonate (<30 days)	All children supported with VA ECMO for cardiac support between Jan 2002 and April, 2013	Werho, 2015	Retrospective review of ELSO registry between Jan 2002 and April 2013	Multivariate	Stroke	OR 1.77 (95% CI 1.32-2.36)	<0.01

Age at ECMO cannulation >48 hours	104 children with ICH from ELSO	Hardart, 1999	Retrospective ELSO review between 1992-1995	Univariate	ICH	RR 0.71	<0.01
Age at ECMO initiation (h, SD)	113 children with ICH versus 859 without	Goodwin, 1995	Retrospective ELSO review between 1986 and 1988	Means	ICH	44.5 (SD 3.6) vs 56.2 (SD 1.8)	<0.01
Age at start ECMO (h, SD)	25 newborns with ICH vs 40 control patients	De Mol, 2008	Retrospective matched case control study	Means	ICH	35 (SD 30) vs 53 (SD 64)	0.20
Age, gestational	752 neonates with gestational age between 29 and 31 weeks (n=243) versus neonates born after 34 weeks of gestation (n=509)	Church, 2017	Retrospective ELSO review between 1976 and 2008	Multivariate	ICH	OR 0.79 (95%CI 0.64–0.98)	0.03
Age, gestational	1898 children with congenital heart disease	Polito, 2015	Retrospective ELSO review between 2005-2010	Means	Stroke/ICH	Stroke/ICH: 38 (IQR 36 - 40)<br>No stroke/ICH: 38 (IQR 35 - 40)	0.01
Age, gestational (weeks)	752 neonates with gestational age between 29 and 31 weeks (n=243) versus neonates born after 34 weeks of gestation (n=509)	Church, 2017	Retrospective ELSO review between 1976 and 2008	Means	Cerebral infarct	Cerebral infarct: 33.36 (SD 0.08),<br>no cerebral infarct 33.49 (SD 0.04)	0.14

Age, gestational (n (%))	752 neonates with gestational age between 29 and 31 weeks (n=243) versus neonates born after 34 weeks of gestation (n=509)	Church, 2017	Retrospective ELSO review between 1976 and 2008	Means	Cerebral infarct	29–33 weeks: 53 (22%)<br>34 weeks: 79 (16%)	0.034
Age, gestational, weeks (SD)	113 children with ICH versus 859 without	Goodwin, 1995	Retrospective ELSO review between 1986 and 1988	Means	ICH	37.8 weeks (SD 0.2) vs 39.2 weeks (SD 0.1)	<0.01
Age, gestational (<34 weeks, 34-36 weeks, 36-38 weeks)	104 children with ICH from ELSO	Hardart, 1999	Retrospective ELSO review between 1992-1995	Means	ICH	<34 weeks OR 12.1 (95% CI 6.6-22.0), 34-36 weeks OR 4.1 (95% CI 2.9-5.8), 36-38 weeks OR 2.1 (95% CI 1.6-2.8)	<0.01
Age, gestational (weeks)	32 infants with PPHN who required ECMO	Doymaz, 2015	Retrospective chart review	Means	ICH	ICH 38.1 weeks (SD 1.2), no ICH 38.3 weeks (SD 1.8)	0.743
Age, gestational (weeks)	25 newborns with ICH vs 40 control patients	De Mol, 2008	Retrospective matched case control study	Means	ICH	ICH 38 (SD 3), no ICH 39 (SD 2)	0.64
Age, postconceptional (weeks)	752 neonates with gestational age between 29 and 31 weeks (n=243) versus neonates born after 34 weeks of gestation (n=509)	Church, 2017	Retrospective ELSO review between 1976 and 2008	Multivariate	ICH	OR 0.82	0.02

Age postconceptional (weeks)	185 patients born at <37 weeks with ICH vs 1194 without	Hardart, 2004	Retrospective ELSO review between 1992-2000	Multivariate	ICH	<34 weeks OR 2.31 (95% CI 1.39-3.85)<br>>34-35 weeks OR 1.36 (0.86-2.14)<br>>35-36 weeks OR 1.10 (0.72-1.62)	<0.01<br>NS<br>NS
APGAR <3 at 1 min (n (%))	113 children with ICH versus 859 without	Goodwin, 1995	Retrospective ELSO review between 1986 and 1988	Means	ICH	ICH 28 (24.8%), no ICH 36 (4.2%)	NS
APGAR <5 at 5 min (n (%))	113 children with ICH versus 859 without	Goodwin, 1995	Retrospective ELSO review between 1986 and 1988	Means	ICH	25 (22.1%) vs 26 (3.0%)	NS
APGAR at 1 min, mean	25 newborns with ICH vs 40 control patients	De Mol, 2008	Retrospective matched case control study	Means	ICH	6 (SD 2.6) vs 6.1 (SD 2.5)	0.94
APGAR at 1 min	752 neonates with gestational age between 29 and 31 weeks (n=243) versus neonates born after 34 weeks of gestation (n=509)	Church, 2017	Retrospective ELSO review between 1976 and 2008	Multivariate	ICH	OR 0.93 (95% CI 0.86–1.005)	0.07
APGAR at 1 min	752 neonates with gestational age between 29 and 31 weeks (n=243) versus neonates born after 34 weeks of gestation (n=509)	Church, 2017	Retrospective ELSO review between 1976 and 2008	Means	Cerebral infarction	Cerebral infarction 5.06 (SD 0.23), no cerebral infarction 4.94 (SD 0.11)	0.62 in total, 45 without circuit change, 17 with circuit change

APGAR at 5 min, mean (SD)	25 newborns with ICH vs 40 control patients	De Mol, 2008	Retrospective matched case control study	Means	ICH	7 (SD 2) vs 7.1 (SD 2.1)	0.82
Baseline ventilation (home ventilator or tracheostomy	514 children	Dalton, 2017	Retrospective analysis	Multivariate	Thrombotic events	RR 0.11 (95% CI 0.02-0.79)	0.025
Chromosomal abnormalities (n (%))	1898 children with congenital heart disease	Polito, 2015	Retrospective ELSO review between 2005-2010	Means	Stroke/ICH	Stroke/ICH: 1 (<1%)<br>No stroke/ICH: 39 (2%)	0.03
Genetic anomaly	All children supported with VA ECMO for cardiac support between Jan 2002 and April, 2013	Werho, 2015	Retrospective review of ELSO registry between Jan 2002 and April 2013	Multivariate	Stroke	OR 0.66 (95% CI 0.38-1.15)	0.14
Non cardiac congenital anomalies (n (%))	1898 children with congenital heart disease	Polito, 2015	Retrospective ELSO review between 2005-2010	Means	Stroke/ICH	Stroke/ICH: 108 (39%)<br>No stroke/ICH: 579 (36%)	0.21
Other complications cerebral infarct, absence of	752 neonates with gestational age between 29 and 31 weeks (n=243) versus neonates born after 34 weeks of gestation (n=509)	Church, 2017	Retrospective ELSO review between 1976 and 2008	Univariate	ICH		<0.01
Other complications Cerebral infarct (n (%))	62 in total, 45 without circuit change, 17 with circuit change	Irby, 2014	Retrospective singe institution study.	Means	Circuit change	No circuit change: no stroke 40 (89%), stroke 5 (11%)<br>Circuit change: no stroke 17 (100), stroke 0(0)	0.310

Other complications, cerebral infarction	752 neonates with gestational age between 29 and 31 weeks (n=243) versus neonates born after 34 weeks of gestation (n=509)	Church, 2017	Retrospective ELSO review between 1976 and 2008	Multivariate	ICH	OR 0.16 (95% CI 0.07 – 0.39)	<0.01
Other complications Cerebral infarction (n (%))	752 neonates with gestational age between 29 and 31 weeks (n=243) versus neonates born after 34 weeks of gestation (n=509)	Church, 2017	Retrospective ELSO review between 1976 and 2008	Means	ICH	ICH 6 (5%), no ICH 126 (95%)	<0.01
Other complications, ICH (n (%))	62 in total, 45 without circuit change, 17 with circuit change	Irby, 2014	Retrospective singe institution study.	Means	Circuit change	No circuit change: no ICH 38 (84%), ICH 7 (16%)<br>Circuit change: no ICH 16 (94%), ICH 1 (16%)	0.427
Other complications ICH	752 neonates with gestational age between 29	Church, 2017	Retrospective ELSO review between 1976 and 2008	Univariate	Cerebral infarct	-	<0.01

	and 31 weeks (n=243) versus neonates born after 34 weeks of gestation (n=509)						
Other complications ICH (n (%))	752 neonates with gestational age between 29 and 31 weeks (n=243) versus neonates born after 34 weeks of gestation (n=509)	Church, 2017	Retrospective ELSO review between 1976 and 2008	Means	Cerebral infarct	Cerebral infarction 6 (5%), no cerebral infarction 129 (21%)	<0.01
Other complications, dialysis (n (%))	752 neonates with gestational age between 29 and 31 weeks (n=243) versus neonates born after 34 weeks of gestation (n=509)	Church, 2017	Retrospective ELSO review between 1976 and 2008	Means	Cerebral infarct	Cerebral infarction 28 (15%), no cerebral infarction 162 (85%)	0.24
Other complications, dialysis (n (%))	752 neonates with gestational age between 29 and 31 weeks (n=243) versus neonates born after 34 weeks of gestation (n=509)	Church, 2017	Retrospective ELSO review between 1976 and 2008	Means	ICH	ICH 36 (19%), no ICH 154 (81%)	0.68

Other complications, dialysis	62 in total, 45 without circuit change, 17 with circuit change	Irby, 2014	Retrospective singe institution study.	Means	Circuit change	No dialysis: no circuit change 27 (60), Circuit change 8 (18)<br>Circuit change: no circuit change 17 (100), Circuit change 0 (0)	0.310
Chronic diagnosis: neurologic, other chronic condition	514 children	Dalton, 2017	Retrospective analysis	Multivariate	Thrombotic events	RR 0.51 (0.23-1.13)	0.023
CPR before ECMO (n (%))	25 newborns with ICH vs 40 control patients	De Mol, 2008	Retrospective matched case control study	Means	ICH	ICH 3 (12.5%), no ICH 3 (7.5%)	0.51
CPR before ECMO	104 children with ICH from ELSO	Hardart, 1999	Retrospective ELSO review between 1992-1995	Univariate	ICH	RR 1.9	<0.01
Pre ECMO cardiac arrest (n (%))	2617 children with respiratory failure	Rollins, 2012	Retrospective ELSO between 1993 and 2007	Means	CNS injury	ICH 44 (19%), no ICH 264 (11%)	<0.05
Pre-ECMO arrest (n (%))	752 neonates with gestational age between 29 and 31 weeks (n=243) versus neonates born after 34 weeks of gestation (n=509)	Church, 2017	Retrospective ELSO review between 1976 and 2008	Means	ICH	ICH 27 (25%), no ICH 80 (75%)	0.03
Pre-ECMO arrest (n (%))	1898 children with congenital heart disease	Polito, 2015	Retrospective ELSO review	Multivariate	Stroke/ICH	OR 1.6 (95% CI 1.2–2.1)	<0.01

			between 2005-2010				
Pre-ECMO arrest (n (%))	752 neonates with gestational age between 29 and 31 weeks (n=243) versus neonates born after 34 weeks of gestation (n=509)	Church, 2017	Retrospective ELSO review between 1976 and 2008	Means	Cerebral infarction	ICH 20 (19%), no ICH 87 (81%)	0.74
Epinephrine use	104 children with ICH from ELSO	Hardart, 1999	Retrospective ELSO review between 1992-1995	Multivariate	ICH	OR 1.9 (95% CI 1.5-2.5)	<0.01
Gender, male (n (%))	113 children with ICH versus 859 without	Goodwin, 1995	Retrospective ELSO review between 1986 and 1988	Means	ICH	IC 58% vs no ICH 59%	NS
Gender, male(n (%))	36 cases (ICH & stroke) vs 36 controls (no ICH/stroke)	Anton-Martin, 2017	Retrospective single center individually matched case-control study	Means	ICH & stroke	Cases: 12 (33.3%)<br>Controls: 18 (50%)	0.23
Gender, female (n (%))	24 patients on whom TEG was performed during ECMO support between 9/1/2011 to 12/31/2012	Saini, 2016	Retrospective single-center chart review	Means	Severe bleeding	No severe bleeding: 9 (64%)<br>Severe bleeding: 5 (50%)	0.57
Gender, female (n (%))	164 children	Muensterer, 2011	Retrospective singe center study	Means	Circuit change	No circuit change: 43%<br>Circuit change:40%	0.71

Gender (F/M)	32 infants with PPHN who required ECMO	Doymaz, 2015	Retrospective chart review	Means	ICH	ICH 4/7, no ICH 5/16	0.453
Gender, male (n (%))	1898 children with congenital heart disease	Polito, 2015	Retrospective ELSO review between 2005-2010	Means	Stroke/ICH	Stroke/ICH: 170 (62%)<br>No stroke/ICH: 951 (58%)	0.41
Gender, male (n (%))	104 children with ICH from ELSO	Hardart, 1999	Retrospective ELSO review between 1992-1995	Univariate	ICH	RR 1.3	<0.05
Gender, male (n (%))	2617 children with respiratory failure	Rollins, 2012	Retrospective ELSO between 1993 and 2007	Means	CNS injury	CNS injury: 105 (45%)<br>No CNS injury: 1173 (49%)	NS
Gender (M/F)	25 newborns with ICH vs 40 control patients	De Mol, 2008	Retrospective matched case control study	Means	ICH	ICH 17/7, no ICH 28/12	0.93
Gender, male (n (%))	62 in total, 45 without circuit change, 17 with circuit change	Irby, 2014	Retrospective singe institution study.	Means	Circuit change	No circuit change: 25 (55%), female 20 (44%)<br>One circuit change: 9 (75%) Multiple circuit changes: 3 (60%)	0.333
Maximum heart rate (BPM)	32 infants with PPHN who required ECMO	Doymaz, 2015	Retrospective chart review	Means	ICH	169.2 (SD 20.9), 176.9 (SD 15.7)	0.301
Minimum heart rate (BPM)	32 infants with PPHN who required ECMO	Doymaz, 2015	Retrospective chart review	Means	ICH	ICH 105.6 (SD 24.2), no ICH 101.7 (SD 14.5)	0.626
Other complications, Infection (n (%))	752 neonates with gestational age between 29 and 31 weeks	Church, 2017	Retrospective ELSO review between 1976 and 2008	Means	ICH	ICH 15 (22%), no ICH 52 (78%)	0.32

	(n=243) versus neonates born after 34 weeks of gestation (n=509)						
Other complications pre-ECMO infection (n (%))	2617 children with respiratory failure	Rollins, 2012	Retrospective ELSO between 1993 and 2007	Means	CNS injury	ICH 131 (56%), no ICH 264 (11%)	<0.05
Other complications Pre-ECMO infection	2617 children with respiratory failure	Rollins, 2012	Retrospective ELSO between 1993 and 2007	Multivariate	CNS injury	OR 1.7 (95% CI 1.3-2.2)	Not mentioned
Other complications Infection (n (%))	752 neonates with gestational age between 29 and 31 weeks (n=243) versus neonates born after 34 weeks of gestation (n=509)	Church, 2017	Retrospective ELSO review between 1976 and 2008	Means	Cerebral infarction	Cerebral infarction 17 (25%), no cerebral infarction 50 (75%)	0.08

Hypertension index (mean)	25 children	Sell, 1986	retrospective review of ECMO patients between Jan 1984 and Nov 1985.	Means	ICH	No ICH 0.1 (SD 0.12) ICH 0.37 (SD 0.28)	<0.05
Mean arterial pressure in first 24 hours, coefficient of variation	25 newborns with ICH vs 40 control patients	De Mol, 2008	Retrospective matched case control study	Means	ICH	ICH 0.13 (SD 0.07), no ICH 0.15 (SD 0.06)	0.42
Mean arterial pressure in first 24 hours (mean)	25 newborns with ICH vs 40 control patients	De Mol, 2008	Retrospective matched case control study	Means	ICH	ICH 52 (SD 8), no ICH 53 (SD 7)	0.63
Minimum mean arterial pressure	32 infants with PPHN who required ECMO	Doymaz, 2015	Retrospective chart review	Means	ICH	ICH 40.09 (SD 6.85), no ICH 36.52 (SD 5.01)	0.148
Mean arterial pressure in first 8 hours, coefficient of variation	25 newborns with ICH vs 40 control patients	De Mol, 2008	Retrospective matched case control study	Means	ICH	ICH 0.13 (0.06), no ICH 0.14 (0.06)	0.60
Mean arterial pressure in first 8 hours (mean)	25 newborns with ICH vs 40 control patients	De Mol, 2008	Retrospective matched case control study	Means	ICH	ICH 53 (SD 10), no ICH 56 (SD7)	0.24
Blood pressure<br>Systolic BP,<br>Diastolic BP<br>Mean BP<br>(mmHg)	1898 children with congenital heart disease	Polito, 2015	Retrospective ELSO review between 2005-2010	Means	Stroke/ICH	Stroke/ICH:<br>47 (IQR 30 - 71)<br>30 (IQR 18 - 48)<br>35 (IQR 22 - 55)<br>No stroke/ICH:<br>50 (IQR 34 - 70)<br>31 (IQR 20 - 45)<br>38 (IQR 26 - 52)	 0.03<br>0.35<br>0.04
Maximum mean arterial pressure (mmHg)	32 infants with PPHN who	Doymaz, 2015	Retrospective chart review	Means	ICH	ICH 69.36 (SD 4.34), no ICH 64.19 (SD 4.86)	<0.01

	required ECMO						
Other complications Mechanical complications (n (%))	752 neonates with gestational age between 29 and 31 weeks (n=243) versus neonates born after 34 weeks of gestation (n=509)	Church, 2017	Retrospective ELSO review between 1976 and 2008	Means	Cerebral infarction	Cerebral infarction 53 (14%), no cerebral infarction 318 (86%)	0.02
Other complications Hemorrhage cannulation (n (%))	2617 children with respiratory failure	Rollins, 2012	Retrospective ELSO between 1993 and 2007	Means	CNS injury	ICH 40 (17%)<br>No ICH 217 (9%)	NS
Other complications Mechanical complications Hemolysis (n (%))	2617 children with respiratory failure	Rollins, 2012	Retrospective ELSO between 1993 and 2007	Means	CNS injury	CNS injury 25 (9%), no CNS injury 217 (9%)	NS
Other complications Mechanical complications Cannula problems (n (%))	2617 children with respiratory failure	Rollins, 2012	Retrospective ELSO between 1993 and 2007	Means	CNS injury	ICH 40 (17%), no ICH 364 (16%)	NS
Other complications Mechanical complications (n (%))	752 neonates with gestational age between 29 and 31 weeks (n=243) versus neonates born after 34 weeks	Church, 2017	Retrospective ELSO review between 1976 and 2008	Means	ICH	ICH 59 (16%), no ICH 2132 (84%)	0.15

<table>
<tr><td></td><td>of gestation
(n=509)</td><td></td><td></td><td></td><td></td><td colspan="2"></td><td></td></tr>
<tr><td>Pre-ECMO narcotic use (n (%))</td><td>752 neonates with gestational age between 29 and 31 weeks (n=243) versus neonates born after 34 weeks of gestation (n=509)</td><td>Church, 2017</td><td>Retrospective ELSO review between 1976 and 2008</td><td>Means</td><td>Cerebral infarction</td><td colspan="2">Cerebral infarction 58 (12%), no cerebral infarction 407 (88%)</td><td><0.01</td></tr>
<tr><td>Pre-ECMO narcotic use (n (%))</td><td>752 neonates with gestational age between 29 and 31 weeks (n=243) versus neonates born after 34 weeks of gestation (n=509)</td><td>Church, 2017</td><td>Retrospective ELSO review between 1976 and 2008</td><td>Means</td><td>ICH</td><td colspan="2">ICH 91 (20%), 374 (80%)</td><td>0.14</td></tr>
<tr><td>Organ failure index on day of ECMO initiation</td><td>514 children</td><td>Dalton, 2017</td><td>Retrospective analysis</td><td>Multivariate</td><td>Daily bleeding event</td><td colspan="2">1: RR 1.00 (0.65-1.53)
2: Reference
3: RR 1.28 (1.08-1.51)
4-5: RR 1.47 (1.09-1.97)</td><td>0.013</td></tr>
<tr><td>Pre-ECMO
Ventilator rate
PIP
PEEP
MAP
FiO2</td><td>1898 children with congenital heart disease</td><td>Polito, 2015</td><td>Retrospective ELSO review between 2005-2010</td><td>Means</td><td>Stroke/ICH</td><td>Stroke/ICH:
30 (IQR 10 - 50)
25 (IQR 18 - 35)
5 (IQR 4 - 7)
11 (IQR 8 - 18)
1 (IQR 0.25 - 1)</td><td>No stroke/ICH:
28 (IQR 12 - 45)
25 (IQR 19 - 35)
5 IQR (3 - 8)
11 (IQR 8 - 18)
1 (IQR 0.21 - 1)</td><td>
0.22
0.99
0.98
0.60
0.38</td></tr>
<tr><td>$PaCO_2$</td><td>113 children with ICH versus 859 without</td><td>Goodwin, 1995</td><td>Retrospective ELSO review between 1986 and 1988</td><td>Means</td><td>ICH</td><td colspan="2">ICH 48.1 (SD 2.4), no ICH 39.6 (SD 0.7)</td><td><0.01</td></tr>
</table>

$PaCO_2$	2617 children with respiratory failure	Rollins, 2012	Retrospective ELSO between 1993 and 2007	Means	CNS injury	ICH 64 (IQR 46-87)<br>No ICH 55 (IQR 43-75)	<0.05
$PaCO_2$	1898 children with congenital heart disease	Polito, 2015	Retrospective ELSO review between 2005-2010	Means	Stroke/ICH	Stroke/ICH: 48 (IQR 28 - 79)<br>No stroke/ICH: 48 (IQR 30 - 77)	<0.01
$PaCO_2$ >45mmhg worst arterial gas	25 newborns with ICH vs 40 control patients	De Mol, 2008	Retrospective matched case control study	Univariate	ICH	OR 3.3 (95% CI 1.0-10.9)	Not mentioned
$PaCO_2$ >50mmhg	104 children with ICH from ELSO	Hardart, 1999	Retrospective ELSO review between 1992-1995	Univariate	ICH	RR 1.8	<0.01
$PaCO_2$ Highest	32 infants with PPHN who required ECMO	Doymaz, 2015	Retrospective chart review	Means	ICH	ICH 65.90 (SD 27.9), no ICH 61 (SD 17.5)	0.601
$PaCO_2$ Lowest	32 infants with PPHN who required ECMO	Doymaz, 2015	Retrospective chart review	Means	ICH	ICH 32.73 (SD 7.02), no ICH 30.10 (SD 6.6)	0.318
PaO_2	113 children with ICH versus 859 without	Goodwin, 1995	Retrospective ELSO review between 1986 and 1988	Means	ICH	ICH 34.7 (SD 1.8), no ICH 40.0 (SD 1.0)	<0.01
PaO_2	104 children with ICH from ELSO	Hardart, 1999	Retrospective ELSO review between 1992-1995	Univariate	ICH	20-<40 RR 1.5<br>40-<60 RR 1.1	NS<br>NS
PaO_2	1898 children with	Polito, 2015	Retrospective ELSO review	Means	Stroke/ICH	Stroke/ICH: 40 (IQR 21 - 178)<br>No stroke/ICH: 42 (IQR 23 - 219)	<0.01

	congenital heart disease		between 2005-2010				
PaO_2 Highest	32 infants with PPHN who required ECMO	Doymaz, 2015	Retrospective chart review	Means	ICH	ICH 189.7 (SD 61.7), no ICH 179.4 (SD 83.9)	0.695
PaO_2 Lowest	32 infants with PPHN who required ECMO	Doymaz, 2015	Retrospective chart review	Means	ICH	ICH 52.2 (SD 16.7), 45.6 (SD 16.2)	0.298
PaO_2 <50 mmHg worst arterial gas	25 newborns with ICH vs 40 control patients	De Mol, 2008	Retrospective matched case control study	Univariate	ICH	OR 5.3 (95% CI 1.5-19.0)	Not mentioned
Patient placed directly on ECMO from CPB	514 children	Dalton, 2017	Retrospective analysis	Multivariate	Daily bleeding event	RR 1.76 (95% CI 1.45-2.13)	<0.01
pre ECMO mechanical support	All children supported with VA ECMO for cardiac support between Jan 2002 and April, 2013	Werho, 2015	Retrospective review of ELSO registry between Jan 2002 and April 2013	Multivariate	Stroke	OR 2.17 (0.98-4.82)	0.06
CPB time <132 min	All children supported with VA ECMO for cardiac support between Jan 2002 and April, 2013	Werho, 2015	Retrospective review of ELSO registry between Jan 2002 and April 2013	Multivariate	Stroke	OR 1.27 (95% CI 0.98-1.64)	0.07
Race, white	104 children with ICH from ELSO	Hardart, 1999	Retrospective ELSO review between 1992-1995	Univariate	ICH	RR 1.8	<0.01

Race	62 in total, 45 without circuit change, 17 with circuit change	Irby, 2014	Retrospective singe institution study.	Means	Circuit change	No circuit change: Caucasian 22 (49%), African American 16 (36%), other 7 (16%)<br>One circuit change: Caucasian 4 (33%) African American 6 (50%), other 2 (17%)<br>Multivariate: Caucasian 3 (60%), African American 2 (40%), other 0	0.798
Race white<br><br>Nonwhite	1898 children with congenital heart disease	Polito, 2015	Retrospective ELSO review between 2005-2010	Means	Stroke/ICH	Stroke/ICH: 157 (57%)<br>No stroke/ICH: 985 (61%) Stroke/ICH: 116 (43%)<br>No stroke/ICH: 640 (39%)	0.33
Race (n (%))	36 cases (ICH & stroke) vs 36 controls (no ICH/stroke)	Anton-Martin, 2017	Retrospective single center individually matched case-control study	Means	ICH & stroke	Cases: African American 10 (27.8%), Asian 1 (2.7%), Caucasian 14 (39%), Hispanic 9 (25%), other 2 (5.5%)<br><br>Controls: African American 12 (33.3%), Asian 0 (0%), Caucasian 10 (27.8%), Hispanic 13 (36.2%), other 1 (2.7%)	0.57
Other complications Seizures	752 neonates with gestational age between 29 and 31 weeks (n=243) versus neonates born after 34 weeks of gestation (n=509)	Church, 2017	Retrospective ELSO review between 1976 and 2008	Univariate	Cerebral infarct	-	<0.01
Other complications, Seizures (n (%))	752 neonates with gestational age between 29 and 31 weeks (n=243) versus neonates born after 34 weeks	Church, 2017	Retrospective ELSO review between 1976 and 2008	Means	ICH	ICH 24 (26%), no ICH 68 (74%)	0.03

	of gestation (n=509)						
Other complications Any hemorrhage (n (%))	62 in total, 45 without circuit change, 17 with circuit change	Irby, 2014	Retrospective singe institution study.	Means	Circuit change	No circuit change: no hemorrhage 27 (60%), hemorrhage 18 (40%)<br>Circuit change: no hemorrhage 12 (71%), hemorrhage 5 (29%)	0.561
Other complications Bleeding complications (n (%))	752 neonates with gestational age between 29 and 31 weeks (n=243) versus neonates born after 34 weeks of gestation (n=509)	Church, 2017	Retrospective ELSO review between 1976 and 2008	Means	Cerebral infarction	Cerebral infarction 8 (10%), no cerebral infarction 70 (90%)	0.07
Other complications Bleeding complications (n (%))	752 neonates with gestational age between 29 and 31 weeks (n=243) versus neonates born after 34 weeks of gestation (n=509)	Church, 2017	Retrospective ELSO review between 1976 and 2008	Means	ICH	ICH 9 (12%), no ICH 69 (88%)	0.12
Other complications Pulmonary hemorrhage (n (%))	62 in total, 45 without circuit change, 17 with circuit change	Irby, 2014	Retrospective singe institution study.	Means	Circuit change	No circuit change: no pulmonary hemorrhage 43 (96%), pulmonary hemorrhage 2 (4%)<br>Circuit change: no pulmonary hemorrhage 17 (100%), pulmonary hemorrhage 0 (0%)	0.999

Other complications Pulmonary hemorrhage (n (%))	752 neonates with gestational age between 29 and 31 weeks (n=243) versus neonates born after 34 weeks of gestation (n=509)	Church, 2017	Retrospective ELSO review between 1976 and 2008	Means	ICH	ICH 5 (14%), no ICH 31 (86%)	0.52
Other complications Pulmonary hemorrhage (n (%))	752 neonates with gestational age between 29 and 31 weeks (n=243) versus neonates born after 34 weeks of gestation (n=509)	Church, 2017	Retrospective ELSO review between 1976 and 2008	Means	Cerebral infarction	Cerebral infarction 11 (31%), no cerebral infarction 25 (69%)	0.04
Other complications, GI hemorrhage (n (%))	62 in total, 45 without circuit change, 17 with circuit change	Irby, 2014	Retrospective singe institution study.	Means	Circuit change	No circuit change: no GI hemorrhage 45 (100) GI hemorrhage 0 (0)<br>Circuit change: no GI hemorrhage 17 (100), GI hemorrhage 0 (0)	-
Other complications, Surgical/cannula site hemorrhage (n (%))	62 in total, 45 without circuit change, 17 with circuit change	Irby, 2014	Retrospective singe institution study.	Means	Circuit change	No circuit change: no hemorrhage 37 (82%), hemorrhage 8 (18%)<br>Circuit change: no hemorrhage 13 (76%), hemorrhage 4 (24%)	0.721

Other complications, pre ECMO liver insufficiency, (n (%))	2617 children with respiratory failure	Rollins, 2012	Retrospective ELSO between 1993 and 2007	Means	CNS injury	ICH 12 (5%)<br>No ICH 37 (2%)	<0.05
Other complications pre ECMO liver insufficiency, (n (%))	2617 children with respiratory failure	Rollins, 2012	Retrospective ELSO between 1993 and 2007	Multivariate	CNS injury	OR 2.0 (95% CI 1.0-4.5)	Not mentioned
Other complications pre ECMO renal failure (n (%))	2617 children with respiratory failure	Rollins, 2012	Retrospective ELSO between 1993 and 2007	Means	CNS injury	ICH 46 (20%)<br>No ICH 239 (10%)	<0.05
Other complications pre ECMO renal failure (n (%))	2617 children with respiratory failure	Rollins, 2012	Retrospective ELSO between 1993 and 2007	Multivariate	CNS injury	OR 1.7 (95% CI 1.3-2.4)	Not mentioned
Other complications pre ECMO chronic lung disease (n (%))	2617 children with respiratory failure	Rollins, 2012	Retrospective ELSO between 1993 and 2007	Means	CNS injury	ICH 19 (8%)<br>No ICH 229 (10%)	NS
Other complications One ventricle CHD pre ECMO	2617 children with respiratory failure	Rollins, 2012	Retrospective ELSO between 1993 and 2007	Means	CNS injury	ICH 5 (2%)<br>No ICH 33 (1%)	NS
Other complications pre ECMO Two ventricle CHD (n (%))	2617 children with respiratory failure	Rollins, 2012	Retrospective ELSO between 1993 and 2007	Means	CNS injury	ICH 12 (5%)<br>No ICH 176 (7%)	<0.05
Other complications	2617 children with	Rollins, 2012	Retrospective ELSO	Means	CNS injury	ICH 11 (5%)<br>No ICH 42 (2%)	<0.05

Immunodefiency pre ECMO	respiratory failure		between 1993 and 2007				
Other complications Cancer pre ECMO (n (%))	2617 children with respiratory failure	Rollins, 2012	Retrospective ELSO between 1993 and 2007	Means	CNS injury	ICH 14 (6%)<br>No ICH 62 (3%)	<0.05
Other complications Cancer pre ECMO	2617 children with respiratory failure	Rollins, 2012	Retrospective ELSO between 1993 and 2007	Multivariate	CNS injury	OR 2.3 (95% CI 1.3-4.3)	Not mentioned
Other complications Other cardiac complications (n (%))	752 neonates with gestational age between 29 and 31 weeks (n=243) versus neonates born after 34 weeks of gestation (n=509)	Church, 2017	Retrospective ELSO review between 1976 and 2008	Means	ICH	ICH 21 (22%), no ICH 75 (78%)	0.27
Other cardiac complications (n (%))	752 neonates with gestational age between 29 and 31 weeks (n=243) versus neonates born after 34 weeks of gestation (n=509)	Church, 2017	Retrospective ELSO review between 1976 and 2008	Means	Cerebral infarct	Cerebral infarction 25 (26%), no cerebral infarction 75 (74%)	0.02
Other complications cyanotic disease	All children supported with VA ECMO for cardiac	Werho, 2015	Retrospective review of ELSO registry between Jan	Multivariate	Stroke	OR 1.26 (95% CI 0.86-.84)	0.23

	support between Jan 2002 and April, 2013		2002 and April 2013					
Other complications clots: any	All children supported with VA ECMO for cardiac support between Jan 2002 and April, 2013	Werho, 2015	Retrospective review of ELSO registry between Jan 2002 and April 2013	Multivariate	Stroke	OR 1.00 (95% CI 0.77-1.29)		0.97
Pre-ECMO surgery (n (%))	1898 children with congenital heart disease	Polito, 2015	Retrospective ELSO review between 2005-2010	Means	Stroke/ICH	Stroke/ICH: 226 (83%)<br>No stroke/ICH: 1,376 (85%)		0.41
Use of inotropics (n (%))	25 newborns with ICH vs 40 control patients	De Mol, 2008	Retrospective matched case control study	Means	ICH	ICH 21 (88%), no ICH 38 (95%)		0.31
Use of inotropics, pre-ECMO	752 neonates with gestational age between 29 and 31 weeks (n=243) versus neonates born after 34 weeks of gestation (n=509)	Church, 2017	Retrospective ELSO review between 1976 and 2008	Multivariate	ICH	OR 2.00 (95% CI 1.03-3.89)		0.04
Medications<br><br>iNO (n (%))<br>Milrinone (n (%))<br>Ranitidine (n (%))	24 patients on whom TEG was performed during ECMO support between	Saini, 2016	Retrospective single-center chart review	Means	Severe bleeding	No severe bleeding:<br>6 (43%)<br>6 (43%)<br>10 (71%)	Severe bleeding:<br><br>6 (60%)<br>5 (50%)<br><br>9 (90%)	 <br>0.16<br>0.78<br><br>0.27

	9/1/2011 to 12/31/2012						
Pre-ECMO vasoactive support (n (%))	1898 children with congenital heart disease	Polito, 2015	Retrospective ELSO review between 2005-2010	Means	Stroke/ICH	Stroke/ICH: 224 (82%)<br>No stroke/ICH: 1,287 (79%)	0.28
Use of inotropes during ECMO (n (%))	752 neonates with gestational age between 29 and 31 weeks (n=243) versus neonates born after 34 weeks of gestation (n=509)	Church, 2017	Retrospective ELSO review between 1976 and 2008	Means	ICH	ICH 30 (24), no ICH 96 (76)	0.06
Use of inotropes during ECMO (n (%))	752 neonates with gestational age between 29 and 31 weeks (n=243) versus neonates born after 34 weeks of gestation (n=509)	Church, 2017	Retrospective ELSO review between 1976 and 2008	Means	Cerebral infarction	Cerebral infarction 9 (7%), no cerebral infarction 117 (93%)	<0.01
Use of steroids	25 newborns with ICH vs 40 control patients	De Mol, 2008	Retrospective matched case control study	Means	ICH	ICH 3 (12.5%), no ICH 5 (12.5%)	0.82
Weight at birth	752 neonates with gestational age between 29 and 31 weeks (n=243) versus neonates born	Church, 2017	Retrospective ELSO review between 1976 and 2008	Multivariate	ICH	OR 0.59 (95% CI 0.37–0.93)	0.02

	after 34 weeks of gestation (n=509)						
Weight at birth (kg)	1898 children with congenital heart disease	Polito, 2015	Retrospective ELSO review between 2005-2010	Means	Stroke/ICH	Stroke/ICH: 2.9 (IQR 2.2 - 3.6)<br>No stroke/ICH: 3.1 (IQR 2.3 - 3.8)	<0.01
Weight at ECMO (kg)	1898 children with congenital heart disease	Polito, 2015	Retrospective ELSO review between 2005-2010	Means	Stroke/ICH	Stroke/ICH: 3.0 (IQR 2.2 - 3.7)<br>No stroke/ICH: 3.1 (IQR 2.4 - 3.9)	<0.01
Weight <3kg	1898 children with congenital heart disease	Polito, 2015	Retrospective ELSO review between 2005-2010	Multivariate	Stroke/ICH	OR 1.5 (95% CI 1.1-1.9)	Not mentioned
Weight at birth (kg)	752 neonates with gestational age between 29 and 31 weeks (n=243) versus neonates born after 34 weeks of gestation (n=509)	Church, 2017	Retrospective ELSO review between 1976 and 2008	Means	Cerebral infarction	Cerebral infarction: 2.39 (SD 0.03), no cerebral infarction 2.43 (SD 0.02)	0.37
Weight for age Z score (for each 1-point decrease)	All children supported with VA ECMO for cardiac support between Jan 2002 and April, 2013	Werho, 2015	Retrospective review of ELSO registry between Jan 2002 and April 2013	Multivariate	Stroke	OR 1.14 (95% CI 1.04 – 1.25)	<0.01
Weight (kg)	36 cases (ICH & stroke) vs 36 controls (no ICH/stroke)	Anton-Martin, 2017	Retrospective single center individually matched	Means	ICH & stroke	Cases: 4.95 (IQR 2.2 – 83)<br>Controls: 6.55 (IQR 2.6 – 50)	0.81

			case-control study				
Weight in kg, median (IQR)	2617 children with respiratory failure	Rollins, 2012	Retrospective ELSO between 1993 and 2007	Means	CNS injury	CNS injury 7.9 (IQR 4.0 - 15.0)<br>No CNS injury 9.5 (IQR4.2 - 21.1)	<0.05
Weight at cannulation (g)	164 children	Muensterer, 2011	Retrospective singe center study	Means	Circuit change	No circuit change: 3212±580<br>Circuit change: 2988±602	0.02
Weight at birth	113 children with ICH versus 859 without	Goodwin, 1995	Retrospective ELSO review between 1986 and 1988	Means	ICH	ICH 3.05 (SD 0.06), no ICH 3.28 (SD 0.02)	<0.01
Weight at birth	104 children with ICH from ELSO	Hardart, 1999	Retrospective ELSO review between 1992-1995	Univariate	ICH	<2.5kg RR 3.3<br>2.5-3.0 RR 1.9	<0.01<br><0.01
Weight at birth<br>Low birth weight<br>High birth weight (%)	13,642 were greater than 2.0 kg and 663 were <€2.0 kg.	Rozmiarek, 2004	Retrospective ELSO review between 1991 and 2002	Means	ICH	Low birth weight: 5.5%<br>High birth weight: 3.6%	<0.01
Weight at birth (kg)	25 newborns with ICH vs 40 control patients	De Mol, 2008	Retrospective matched case control study	Means	ICH	ICH 3.3 (SD 0.5), no ICH 3.3 (SD 0.5)	0.79
Weight (kg)	32 infants with PPHN who required ECMO	Doymaz, 2015	Retrospective chart review	Means	ICH	ICH 3.3 (SD 0.2), no ICH 3.4 (SD 0.6)	0.602
Weight (kg)	274 patients with 299 ECMO runs. 38 circuit changes	Maul, 2020	Retrospective analysis	Means	Circuit change	No circuit change 14 (SD 21), circuit change 11 (SD 14)	NS

Year ECMO, period 1992-2008	752 neonates with gestational age between 29 and 31 weeks (n=243) versus neonates born after 34 weeks of gestation (n=509)	Church, 2017	Retrospective ELSO review between 1976 and 2008	Univariate	ICH	-	<0.01
Year ECMO, period 1992-2008	752 neonates with gestational age between 29 and 31 weeks (n=243) versus neonates born after 34 weeks of gestation (n=509)	Church, 2017	Retrospective ELSO review between 1976 and 2008	Univariate	Cerebral infarct	-	<0.01
Year ECMO 1993-1997 (n (%))	2617 children with respiratory failure	Rollins, 2012	Retrospective ELSO between 1993 and 2007	Means	CNS injury	CNS injury 34 (15%)<br>No CNS injury 588 (25%)	NS
Year ECMO 1998-2002 (n (%))	2617 children with respiratory failure	Rollins, 2012	Retrospective ELSO between 1993 and 2007	Means	CNS injury	CNS injury 85 (36%)<br>No CNS injury 829 (35%)	NS
Year ECMO 2003-2007 (n (%))	2617 children with respiratory failure	Rollins, 2012	Retrospective ELSO between 1993 and 2007	Means	CNS injury	CNS injury: 114 (49%)<br>No CNS injury 967 (41%)	NS

Hematological risk factors associated with bleeding and thrombosis in pediatric ECMO patients.

Antithrombin	62 in total, 45 without circuit change, 17 with circuit change	Irby, 2014	Retrospective singe institution study.	Means	Circuit change	No circuit change: 72.25 (SD 2.82)<br>Circuit change: 60.42 (SD 2.27)	<0.01
Coagulopathy	104 children with ICH from ELSO	Hardart, 1999	Retrospective ELSO review between 1992-1995	Multivariate	ICH	OR 1.6 (95% CI 1.1-2.2)	<0.01
Pre-ECMO coagulopathy (n (%))	84 with pre-ECMO coagulopathy vs 91 without pre-ECMO coagulopathy	Anton-Martin, 2017	Retrospective single-center chart review	Means	Cannulation site bleed<br>Chest Tube Site<br><br>Intracranial<br><br>Line Site<br><br>Pulmonary<br><br>Other<br><br>Surgical Site<br><br>Gastrointestinal<br><br>Pericardial	With 20 (28.2)<br>Without: 20 (29)<br>With: 11 (15.5)<br>Without: 17 (24.7)<br>With: 8 (11.2)<br>Without: 6 (8.7)<br>With: 9 (12.7)<br>Without: 3 (4.3)<br>With: 9 (12.7)<br>Without: 2 (3)<br>With: 6 (8.5)<br>Without: 3 (4.3)<br>With: 2 (2.8)<br>Without: 8 (11.6)<br>With: 2 (2.8)<br>Without: 5 (7.2)<br>With: 4 (5.6)<br>Without: 5 (7.2)	0.19
DIC (n (%))	752 neonates with gestational age between 29 and 31 weeks (n=243) versus neonates born	Church, 2017	Retrospective ELSO review between 1976 and 2008	Means	Cerebral infarction	Cerebral infarction 3 (19%), no cerebral infarction 13 (81%)	0.90

	after 34 weeks of gestation (n=509)						
DIC (n (%))	752 neonates with gestational age between 29 and 31 weeks (n=243) versus neonates born after 34 weeks of gestation (n=509)	Church, 2017	Retrospective ELSO review between 1976 and 2008	Means	ICH	ICH 5 (31%), no ICH 11 (69%)	0.16
DIC (n (%))	2617 children with respiratory failure	Rollins, 2012	Retrospective ELSO between 1993 and 2007	Means	CNS injury	ICH 20 (9%), no ICH 122 (5%)	<0.05
Cryoprecipitate	145 post-cardiotomy patients on ECMO in excessive and non-excessive bleeding group	Nardell, 2009	Retrospective review of ELSO between Jan 1998 and Jun 2007	Means	Bleeding complications	0-6h: NE 16.0±1.0 E 21.9±3.2<br>7-12h: NE 11.27±1.3 E 23.6±3.7<br>13-24h: NE 24.9±7.1 E 17.5±7.5<br>25-48h: NE 12.9±1.2 E 20.5±9.5<br>49h-5d: NE 22.8±3.6 E 52.0*	p=0.08<br>p=0.02<br>p=0.52<br>p=0.57<br>N/A
Cryoprecipitate volume (mL)	32 infants with PPHN who required ECMO	Doymaz, 2015	Retrospective chart review	Means	ICH	ICH 15.45 (SD 20.18), no ICH 7.85 (SD 21.5)	0.346
Fibrinogen	145 post-cardiotomy patients on ECMO in excessive and	Nardell, 2009	Retrospective review of ELSO between Jan 1998 and Jun 2007	Means	Bleeding complications	0-6h: NE 112±54 E 109±61<br>7-12h: NE 209±81 E 176±73<br>13-24h: NE 195±60 E 127±55	0.7<br>0.2<br>0.02

	non-excessive bleeding group					25-48h: NE 199±75 E 141±96<br>49h-5d: NE 237±106 E 163*	0.2<br>N/A
Maximum fibrinogen	32 infants with PPHN who required ECMO	Doymaz, 2015	Retrospective chart review	Means	ICH	ICH 326 (SD 119) vs no ICH 336 (SD 83.9)	0.809
Minimum fibrinogen	32 infants with PPHN who required ECMO	Doymaz, 2015	Retrospective chart review	Means	ICH	ICH 115 (SD 43.8) no ICH 175 (SD 74.8)	<0.01
Heparin (total units/interval)	145 post-cardiotomy patients on ECMO in excessive and non-excessive bleeding group	Nardell, 2009	Retrospective review of ELSO between Jan 1998 and Jun 2007	Means	Bleeding.	0-6h:<br>NE 208±354<br>E 108±120<br>7-12h:<br>NE 382±647<br>E 371±355<br>13-24h:<br>NE 801±999<br>E 1024±1223<br>25-48h:<br>NE 2121±2303<br>E 2329±1044<br>49h-5d:<br>NE 7400±11000<br>E 3303±607	0.07<br>0.9<br>0.5<br>0.9<br>0.6
Heparin (average heparin infusion rate) (IU/kg)	62 in total, 45 without circuit change, 17 with circuit change	Irby, 2014	Retrospective singe institution study.	Means	Circuit change	No circuit change: 24.69 (SD 1.34)<br>Circuit change: 20.79 (SD 1.11)	<0.01

Heparin dose (units/kg/hr), median	69 neonatal and pediatric ECMO patient	McMichael, 2019	Retrospective single-center review	Means	Any bleed	Bleed: 25 (IQR 17–39)<br>No bleed: 31 (IQR 25–35)	0.15
Heparin dose (units/kg/hr), median	69 neonatal and pediatric ECMO patient	McMichael, 2019	Retrospective single-center review	Means	GI bleed	Bleed: 46 (IQR 32–51)<br>No bleed: 30 (IQR 22–35)	0.05
Heparin dose (units/kg/hr), median	69 neonatal and pediatric ECMO patient	McMichael, 2019	Retrospective single-center review	Means	ICH	Bleed: 26 (IQR 18–32)<br>No bleed: 31 (IQR 23–36)	0.24
Heparin dose (units/kg/hr), median	69 neonatal and pediatric ECMO patient	McMichael, 2019	Retrospective single-center review	Means	Surgical site bleed	Bleed: 23 (IQR 16–43)<br>No bleed: 31 (IQR 23–35)	0.29
Heparin dose (units/kg/hr), median	69 neonatal and pediatric ECMO patient	McMichael, 2019	Retrospective single-center review	Means	Circuit clots	Clots: 32 (IQR 23–38)<br>No clots: 27 (IQR 21–35) 0.18	0.18
Heparin dose (units/kg/hr), median	69 neonatal and pediatric ECMO patient	McMichael, 2019	Retrospective single-center review	Means	Stroke	Stroke: 22 (IQR 16–25)<br>No stroke 31 (IQR 23–36)	0.15
Heparin dose (U/kg/h)	24 patients on whom TEG was performed during ECMO support between 9/1/2011 to 12/31/2012	Saini, 2016	Retrospective single-center chart review	Means	Severe bleeding	No severe bleeding: 40 IQR (30–50)<br>Severe bleeding: 30 (IQR 20–48)	0.04

Infusions >10 in first 24 hours	25 newborns with ICH vs 40 control patients	De Mol, 2008	Retrospective matched case control study	Multivariate	ICH	OR 8.5 (95% CI 1.2-59.2)	0.03
Infusions >300m in first 8 hours	25 newborns with ICH vs 40 control patients	De Mol, 2008	Retrospective matched case control study	Multivariate	ICH	OR 10.0 (95% CI 1.9-83.3)	0.03
Infusions >400mL in first 24 hours	25 newborns with ICH vs 40 control patients	De Mol, 2008	Retrospective matched case control study	Multivariate	ICH	OR 6.4 (95% CI 0.5-77.1)	NS
Infusions >8 infusions in first 8 hours	25 newborns with ICH vs 40 control patients	De Mol, 2008	Retrospective matched case control study	Multivariate	ICH	OR 1.3 (95% CI 1.0-1.6)	0.02
Infusions, every 100mL of volume first 24 hours	25 newborns with ICH vs 40 control patients	De Mol, 2008	Retrospective matched case control study	Multivariate	ICH	OR 1.6 (95% CI 1.0-2.4)	0.04
Infusions, every 100mL of volume first 8 hours	25 newborns with ICH vs 40 control patients	De Mol, 2008	Retrospective matched case control study	Multivariate	ICH	OR 1.6 (95% CI 1.0-2.6)	0.06
Infusions, every single infusion first 24h	25 newborns with ICH vs 40 control patients	De Mol, 2008	Retrospective matched case control study	Multivariate	ICH	OR 1.2 (95% CI 1.0-1.4)	0.04
Infusions, every single infusion first 8 hours	25 newborns with ICH vs 40 control patients	De Mol, 2008	Retrospective matched case control study	Multivariate	ICH	OR 1.3 (95% CI 1.0-1.6)	0.04
Infusions fresh frozen plasma volume (mL)	32 infants with PPHN who required ECMO	Doymaz, 2015	Retrospective chart review	Means	ICH	ICH 107.6 (SD 89.2), no ICH 86.8 (SD 44.15)	0.380

Infusions fresh frozen plasma	145 post-cardiotomy patients on ECMO in excessive and non-excessive bleeding group	Nardell, 2009	Retrospective review of ELSO between Jan 1998 and Jun 2007	Means	Bleeding complications	0-6h:<br>NE 106.6±16.3<br>E 294.5±55.3<br>7-12h:<br>NE 107.9±19.7<br>E 327.2±107.3<br>13-24h:<br>NE 122.2±24.2<br>E 25±79.1<br>25-48h:<br>NE 167.9±49.9<br>E 796.0±686<br>49h-5d:<br>NE 284.9±75.2<br>E **	p=0.002<br><br><br>p=0.07<br><br><br>p=0.13<br><br><br>p=0.53<br><br><br>N/A
Blood products transfused pRBC transfusion (mL/kg)	24 patients on whom TEG was performed during ECMO support between 9/1/2011 to 12/31/2012	Saini, 2016	Retrospective single-center chart review	Means	Severe bleeding	No severe bleeding:<br>9 (0–20<br>Severe bleeding:<br>20 (0-30)	0.10
Blood products transfused PC transfusion (mL/kg)	24 patients on whom TEG was performed during ECMO support between 9/1/2011 to 12/31/2012	Saini, 2016	Retrospective single-center chart review	Means	Severe bleeding	No severe bleeding:<br>0 (0–15)<br>Severe bleeding:<br>8 (0-17.5)	0.86
Blood products transfused FFP transfusion (mL/kg)	24 patients on whom TEG was performed during ECMO support between	Saini, 2016	Retrospective single-center chart review	Means	Severe bleeding	No severe bleeding:<br>0 (0–15)<br>Severe bleeding:<br>4 (0–15)	0.72

	9/1/2011 to 12/31/2012						
Number of platelet transfusions after 24 hours	25 newborns with ICH vs 40 control patients	De Mol, 2008	Retrospective matched case control study	Means	ICH	ICH 2.1 (SD 1.2), no ICH 1.6 (SD 1.2)	0.64
Transfusions packed red blood cells	62 in total, 45 without circuit change, 17 with circuit change	Irby, 2014	Retrospective singe institution study.	Means	Circuit change	No circuit change: 33.40 (4.12)<br>Circuit change: 40.59 (5.78)	0.311
Platelet count	17 in group 1 (PC>10) and 25 in group 2 (PC>20)	Stallion, 1994	Retrospective study from treatment period >100000 platelet count or >200000	Means	Bleeding complications	Group >200.000 platelet count 12% bleeding complications vs 34% from ELSO	<0.01
Platelet count (*10^9/L)	62 in total, 45 without circuit change, 17 with circuit change	Irby, 2014	Retrospective singe institution study.	Means	Circuit change	No circuit change: 152.10 (SD 12.10)<br>Circuit change: 135.09 (SD 10.61)	0.291
Platelet count, standard protocol vs bleeding protocol vs +ACA	164 children	Muensterer, 2011	Retrospective singe center study	Means	Circuit life	Not mentioned	SP vs BP: 0.12<br>SP vs BP+ACA: 0.92
Platelet count	17 in group 1 (PC>10) and 25 in group 2 (PC>20)	Stallion, 1994	Retrospective study from treatment period >100000 platelet count or >200000	Means	ICH	Group >100.000 platelet count 5.9% vs 8% in group	NS

						>200.000 platelet count	
Platelet count	17 in group 1 (PC>10) and 25 in group 2 (PC>20)	Stallion, 1994	Retrospective study from treatment period >100000 platelet count or >200000	Means	Cannula site/wound	Group >100.000 platelet count 23.5% vs 4% in group >200.000 platelet count	NS
Platelet counts, Percentage of low	25 children	Sell, 1986	retrospective review of ECMO patients between Jan 1984 and Nov 1985.	Means	ICH	<100 ICH 57.5% vs 42.1% no ICH<br>>100 ICH 42.5% vs 58% no ICH	<0.05<br><0.05
Minimum platelet level (*10^9/L)	113 children with ICH versus 859 without	Goodwin, 1995	Retrospective ELSO review between 1986 and 1988	Means	ICH	ICH 107 (SD 9), no ICH 95 (SD 4)	0.65
Maximum platelet level (*10^9/L)	113 children with ICH versus 859 without	Goodwin, 1995	Retrospective ELSO review between 1986 and 1988	Means	ICH	ICH 216 (SD 20), no ICH 233 (SD 10)	0.44
Minimum platelet level	32 infants with PPHN who required ECMO	Doymaz, 2015	Retrospective chart review	Means	ICH	37.4 (18.3) vs 60.4 (23.3)	<0.01
Maximum platelet level	32 infants with PPHN who required ECMO	Doymaz, 2015	Retrospective chart review	Means	ICH	116.4 (18.9) vs 140.7 (35.4)	0.017
Platelets (*10^9/L)	145 post-cardiotomy patients on ECMO in excessive and non-excessive bleeding group	Nardell, 2009	Retrospective review of ELSO between Jan 1998 and Jun 2007	Means	Bleeding complications	0-6h:<br>NE 104.8±50<br>E 84.3±41<br>7-12h:<br>NE 127.0±50<br>E 95.6 ±45<br>13-24h:<br>NE 97.7±9.9<br>E 84.2±31.0<br>25-48h:	p= 0.02<br>p= 0.02<br>p= 0.8<br>p= 0.3

						NE 108.0 ±21<br>E 80.7±33<br>49h-5d:<br>NE 104.7±21<br>E 92.4±18	p= 0.5
Volume of platelet (fL)	25 newborns with ICH vs 40 control patients	De Mol, 2008	Retrospective matched case control study	Means	ICH	ICH 85 (SD 48), no ICH 67 (SD 48)	0.18
Platelet volume (fL)	32 infants with PPHN who required ECMO	Doymaz, 2015	Retrospective chart review	Means	ICH	ICH 400.90 (SD 314.8), no ICH 434.30 (SD 407.19)	0.812
Platelet count (*10^9/L)	Non-surgical neonates on VV or VA ECMO with ICH vs without ICH	Grayck, 1995	Retrospective chart review between Jan 91 and Feb 94	Means	ICH	Minimum: ICH 107000 (SD 9000), no ICH 95000 (SD 4000)<br>Maximum: ICH 216000 (SD 20000), no ICH 233000 (SD 10000)	0.65<br>0.44

ECMO/surgical related risk factors for bleeding or thrombosis in pediatric ECMO patients.

Cephalic jugular venous drainage	505 children with ICH from ELSO	Hardart, 1999	Retrospective ELSO review between 1992-1995	Univariate	ICH	OR 0.92 (95% CI 0.66-1.30)	NS
Cephalic jugular venous drainage	104 children with ICH from ELSO	Hardart, 1999	Retrospective ELSO review between 1992-1995	Multivariate	ICH	OR 0.86 (95% CI 0.61-1.22)	NS
Duration of ECMO (h)	32 infants with PPHN who required ECMO	Doymaz, 2015	Retrospective chart review	Means	ICH	ICH 183 (SD 14), no ICH 164 (SD 11)	0.680
Duration of ECMO (h)	25 newborns with ICH vs 40 control patients	De Mol, 2008	Retrospective matched case control study	Means	ICH	ICH 155 (SD 76) vs 171 (SD 81)	0.43
Duration of ECMO>167 hours	All children supported with VA ECMO for cardiac support between Jan 2002 and April, 2013	Werho, 2015	Retrospective review of ELSO registry between Jan 2002 and April 2013	Multivariate	Stroke	OR 1.38 (95% CI 1.06-1.78)	0.02
Duration of ECMO (days)	24 patients on whom TEG was performed during ECMO support between 9/1/2011 to 12/31/2012	Saini, 2016	Retrospective single-center chart review	Means	Severe bleeding	No severe bleeding: 7 (IQR 6–10)<br>Severe bleeding: 8 (IQR 5–9)	0.99
Duration of ECMO (mean)	274 patients with 299 ECMO runs. 38 circuit changes	Maul, 2020	Retrospective analysis	Means	Circuit change	No circuit change 113 (SD 102), circuit change 383 (SD512)	<0.05
Duration of ECMO (h)	2617 children with respiratory failure	Rollins, 2012	Retrospective ELSO between 1993 and 2007	Means	CNS injury	ICH: 176 (IQR 88 - 326)<br>No ICH 201 (IQR 107 - 356)	<0.05
Duration of ECMO (hours)	164 children	Muensterer, 2011	Retrospective singe center study	Means	Circuit change	No circuit change 195 (SD 108)	<0.01

						Circuit change 504 (SD 224)	
Duration of ECMO (h)	1898 children with congenital heart disease	Polito, 2015	Retrospective ELSO review between 2005-2010	Means	Stroke/ICH	Stroke/ICH: 114 (IQR 34 - 351)<br>No stroke/ICH: 110 (IQR 39 - 295)	0.61
Duration of ECMO (h)	36 cases (ICH & stroke) vs 36 controls (no ICH/stroke)	Anton-Martin, 2017	Retrospective single center individually matched case-control study	Means	ICH & stroke	Cases: 154 (IQR 25–1047)<br>Controls: 176 (IQR 50–1702)	0.48
ECMO indication (respiratory is reference)<br>Cardiac<br>ECPR	514 children	Dalton, 2017	Retrospective analysis	Multivariate	Daily bleeding event	RR 1.34 (95% CI 1.11-1.63)<br>RR 1.52 (95% CI 1.16 -1.98)	<0.01
ECMO indication (n (%))	36 cases (ICH & stroke) vs 36 controls (no ICH/stroke)	Anton-Martin, 2017	Retrospective single center individually matched case-control study	Means	ICH & stroke	Cases: pulmonary 18 (50%), cardiac 4 (11.2%), ECPR 14 (38.8%)<br>Controls: pulmonary 25 (69.4%), cardiac 5 (13.9%), ECPR 6 (16.7)	0.1
ECMO indication<br>Cardiac<br>Pulmonary<br>ECPR<br>(n (%))	1898 children with congenital heart disease	Polito, 2015	Retrospective ELSO review between 2005-2010	Means	Stroke/ICH	Stroke/ICH:<br>13 (5%)<br>216 (79%)<br>44 (16%)<br>No stroke/ICH:<br>111 (7%)<br>1,289 (79%) 225 (14%)	0.30
ECMO indication<br>ECPR<br>(n (%))	62 in total, 45 without circuit change, 17 with circuit change	Irby, 2014	Retrospective singe institution study.	Means	Circuit change	No circuit change:<br>14 (31%)<br>One circuit change:<br>2 (17%)<br>Multiple circuit changes:<br>0	0.327

ECMO indication septic shock (n (%))	62 in total, 45 without circuit change, 17 with circuit change	Irby, 2014	Retrospective singe institution study.	Means	Circuit change	No circuit change: 2 (4%)<br>One circuit change: 0<br>Multiple circuit changes: 0	0.327
ECMO indication neonatal respiratory failure (n (%))	62 in total, 45 without circuit change, 17 with circuit change	Irby, 2014	Retrospective singe institution study.	Means	Circuit change	No circuit change: 13 (29%)<br>Circuit change: 6 (50%)<br>Multiple circuit changes: 3 (60%)	0.327
ECMO indication pediatric respiratory failure (n (%))	62 in total, 45 without circuit change, 17 with circuit change	Irby, 2014	Retrospective singe institution study.	Means	Circuit change	No circuit change: 3 (7%)<br>Circuit change: 0<br>Multiple circuit changes: 1 (20%)	0.327
ECMO indication cardiac/postoperative support (n (%))	62 in total, 45 without circuit change, 17 with circuit change	Irby, 2014	Retrospective singe institution study.	Means	Circuit change	NC: 5 (11%)<br>One: 3 (2%5)<br>Multiple circuit changes: 1 (20%)	0.327
ECMO indication cardiac/bridge to transplant (n (%))	62 in total, 45 without circuit change, 17 with circuit change	Irby, 2014	Retrospective singe institution study.	Means	Circuit change	NC: 1 (2%)<br>One: 1 (8%)<br>Multiple circuit changes: 0 (0%)	0.327
ECMO indication cardiac/other (n (%))	62 in total, 45 without circuit	Irby, 2014	Retrospective singe	Means	Circuit change	NC: 7 (16%)<br>One: 0 (0%)	0.327

	change, 17 with circuit change		institution study.			Multiple circuit changes: 0 (0%)		
ECMO indication, Respiratory (%)	274 patients with 299 ECMO runs. 38 circuit changes	Maul, 2020	Retrospective analysis	Means	Circuit change	No circuit change 38%, Circuit change 63%		<0.05
ECMO indication: cardiac and ECPR (%)	274 patients with 299 ECMO runs. 38 circuit changes	Maul, 2020	Retrospective analysis	Means	Circuit change	Circuit change 63% vs Circuit change 37%		<0.05
Primary diagnosis of sepsis	104 children with ICH from ELSO	Hardart, 1999	Retrospective ELSO review between 1992-1995	Multivariate	ICH	OR 1.8 (95% CI 1.4-2.4)		<0.01
Primary diagnosis of sepsis	185 patients born at <37 weeks with ICH vs 1194 without	Hardart, 2004	Retrospective ELSO review between 1992-2000	Multivariate	ICH	OR 1.78 (95% CI 1.24-2.56)		<0.01
Primary diagnosis:<br>- Other<br>- Respiratory	514 children	Dalton, 2017	Retrospective analysis	Multivariate	Thrombotic events	RR 1.22 (95% CI 0.52-2.89)<br>RR 0.67 (95% CI 0.50-0.88)		0.018
Primary diagnosis of<br>Sepsis/Pneumonia<br>Cardiac<br>RDS/Lung anomalies<br>CDH<br>(n (%))	752 neonates with gestational age between 29 and 31 weeks (n=243) versus neonates born after 34 weeks of gestation (n=509)	Church, 2017	Retrospective ELSO review between 1976 and 2008	means	ICH	Sepsis/pneumonia ICH 34 (22%), no ICH 122 (78%)<br>Cardiac ICH 24 (18%) no ICH 108 (82%)<br>RDS/lung anomaly ICH 38 (15%) no ICH 219 (85%)<br>CDH ICH 39 (19%) no ICH 165 (82%)		0.32
Cardiac diagnosis<br>d-TGA<br>UVH<br>RV obstruction<br>LV obstruction<br>Truncus arteriosus	1898 children with congenital heart disease	Polito, 2015	Retrospective ELSO review between 2005-2010	Means	Stroke/ICH	Stroke/ICH<br><br>25 (9%)<br>100 (36%)<br>40 (15%)<br>35 (13%)	No stroke/ICH<br>157 (10%)<br>535 (33%)<br>222 (13%)<br>202 (12%)	0.16

Transplant/CM/<br>myocarditis<br>TAPVR<br>Others<br>(n (%))						8 (3%) 66 (4%)<br>11 (4%) 71 (5%)<br>16 (6%) 176 (11%)<br>39 (14%) 196 (12%)	
Primary diagnosis<br>CDH<br>(n (%))	164 children	Muensterer, 2011	Retrospective singe center study	Means	Circuit change	No circuit change 20 (17 %)<br>Circuit change 40 (65 %)	< 0.01
Primary diagnosis<br>Meconium aspiration<br>(n (%))	164 children	Muensterer, 2011	Retrospective singe center study	Means	Circuit change	No circuit change 19 (16%) Circuit change 5 (8%)	0.13
Primary diagnosis<br>Sepsis<br>(n (%))	164 children	Muensterer, 2011	Retrospective singe center study	Means	Circuit change	No circuit change 37 (31%)<br>Circuit change 2 (3%)	< 0.01
Primary diagnosis<br>PPHN (n (%))	164 children	Muensterer, 2011	Retrospective singe center study	Means	Circuit change	No circuit change 28 (24%)<br>Circuit change 7 (11%)	0.05
Primary diagnosis<br>Other (n (%))	164 children	Muensterer, 2011	Retrospective singe center study	Means	Circuit change	No circuit change 14 (12 %) Circuit change 8 (13%)	Not described
Primary diagnosis RS virus (n (%))	2617 children with respiratory failure	Rollins, 2012	Retrospective ELSO between 1993 and 2007	Means	CNS injury	ICH 43 (19%)<br>No ICH 43 (19%)	NS
High risk pulmonary diagnosis	2617 children with respiratory failure	Rollins, 2012	Retrospective ELSO between 1993 and 2007	Multivariate	CNS injury	OR 1.4 (95% CI 1.1-2.0)	Not mentioned
Primary diagnosis<br>bacterial pneumonia<br>(n (%))	2617 children with respiratory failure	Rollins, 2012	Retrospective ELSO between	Means	CNS injury	ICH 49 (21%)<br>No ICH 381 (16%)	<0.05

			1993 and 2007				
Primary diagnosis other viral pneumonia (n (%))	2617 children with respiratory failure	Rollins, 2012	Retrospective ELSO between 1993 and 2007	Means	CNS injury	ICH 17 (7%)<br>No ICH 240 (10%)	NS
Primary diagnosis Sepsis ARDS	2617 children with respiratory failure	Rollins, 2012	Retrospective ELSO between 1993 and 2007	Means	CNS injury	ICH 23 (10%)<br>No ICH 157 (7%)	<0.05
Primary diagnosis Aspiration pneumonia (n (%))	2617 children with respiratory failure	Rollins, 2012	Retrospective ELSO between 1993 and 2007	Means	CNS injury	ICH 7 (3%)<br>No ICH 125 (5%)	NS
Primary diagnosis Trauma ARDS (n (%))	2617 children with respiratory failure	Rollins, 2012	Retrospective ELSO between 1993 and 2007	Means	CNS injury	ICH 9 (4%)<br>No ICH 104 (4%)	NS
Primary diagnosis Pertussis (n (%))	2617 children with respiratory failure	Rollins, 2012	Retrospective ELSO between 1993 and 2007	Means	CNS injury	ICH 15 (6%)<br>No ICH 60 (3%)	<0.05
Primary diagnosis Pulmonary hemorrhage (n (%))	2617 children with respiratory failure	Rollins, 2012	Retrospective ELSO between 1993 and 2007	Means	CNS injury	ICH 2 (1%)<br>No ICH 59 (3%)	NS
Primary diagnosis Other ARDS (n (%))	2617 children with respiratory failure	Rollins, 2012	Retrospective ELSO between 1993 and 2007	Means	CNS injury	ICH 2 (1%)<br>No ICH 26 (1%)	NS

Primary diagnosis<br>Other (n (%))	2617 children with respiratory failure	Rollins, 2012	Retrospective ELSO between 1993 and 2007	Means	CNS injury	ICH 66 (28%)<br>No ICH 826 (34%)	NS
Primary diagnosis of<br>Sepsis/Pneumonia<br>Cardiac<br>RDS/Lung anomalies<br>CDH	752 neonates with gestational age between 29 and 31 weeks (n=243) versus neonates born after 34 weeks of gestation (n=509)	Church, 2017	Retrospective ELSO review between 1976 and 2008	Multivariate	Cerebral infarction	OR 0.48 (95% CI 0.24–0.95)<br>OR 0.45 (95% CI 0.26–0.78)<br>OR 0.60 (95% CI 0.32–1.10)	0.04<br><br><0.01<br><br>0.10
Primary diagnosis<br>Pneumonia<br>MAS (%)	113 children with ICH versus 859 without	Goodwin, 1995	Retrospective ELSO review between 1986 and 1988	Means	ICH	ICH 21.9%, no ICH 11.6%<br>ICH 6.7%, no ICH 11.6%,	P < 0.05<br>P <0.05
MAS	514 children	Dalton, 2017	Retrospective analysis	Multivariate	Daily bleeding event	RR 0.54 (95% CI 0.34-0.84)	<0.01
Mode of cannulation ECMO, VV vs VA ECMO	514 children	Dalton, 2017	Retrospective analysis	Multivariate	Thrombotic events	RR 0.62 (95% CI 0.40–0.95)	0.024
Mode of cannulation<br>VA ECMO	2617 children with respiratory failure	Rollins, 2012	Retrospective ELSO between 1993 and 2007	Multivariate	CNS injury	OR 1.6 (1.1-2.3)	Not mentioned
Mode of cannulation (n (%))	2617 children with respiratory failure	Rollins, 2012	Retrospective ELSO between 1993 and 2007	Means	Surgical site bleed	Carotid VA: 1 (11%)<br>Femoral VA : 31 (27%)<br>VV: 98 (10%)	<0.05 carotid vs femoral
Mode of cannulation (n (%))	2617 children with respiratory failure	Rollins, 2012	Retrospective ELSO between	Means	Cannula site bleed	Carotid VA: 209 (14%)<br>Femoral VA : 32 (28%)<br>VV: 285 (19%)	<0.05 car vs VV<br><0.05 car vs fem

			1993 and 2007				
Mode of cannulation, VA ECMO (n (%))	24 patients on whom TEG was performed during ECMO support between 9/1/2011 to 12/31/2012	Saini, 2016	Retrospective single-center chart review	Means	Severe bleeding	No severe bleeding: 10 (71%)<br>Severe bleeding: 9 (90%)	0.17
Mode of cannulation, VA vs VV (n (%))	763 children treated in US centers	Zahraa, 2000	Retrospective ELSO review between 1984 and Jul 1997	Means	Stroke	VA: 14 (2.4%)<br>VV: 3 (1.8%)	1.000
Mode of cannulation, VA vs VV (n (%))	763 children treated in US centers	Zahraa, 2000	Retrospective ELSO review between 1984 and Jul 1997	Means	ICH	VA: 24 (4%)<br>VV: 6 (3.6%)	0.785
Mode of cannulation VA ECMO VV ECMO Others (n (%))	1898 children with congenital heart disease	Polito, 2015	Retrospective ELSO review between 2005-2010	Means	Stroke/ICH	Stroke/ICH:<br>267 (97%)<br>4 (2%)<br>1 (<1%)<br>No stroke/ICH:<br>1,573 (97%)<br>43 (2%)<br>6 (<1%)	0.51
Neck cannulation (n (%))	1898 children with congenital heart disease	Polito, 2015	Retrospective ELSO review between 2005-2010	Means	Stroke/ICH	Stroke/ICH: 93 (34%)<br>No stroke/ICH: 467 (29%)	0.09
Neck cannulation	All children supported with VA ECMO for cardiac support between Jan 2002 and April, 2013	Werho, 2015	Retrospective review of ELSO registry between Jan 2002 and April 2013	Multivariate	Stroke	OR 1.29 (95% CI 0.91-1.81)	0.15
Mode of cannulation (n (%))	2617 children with respiratory failure	Rollins, 2012	Retrospective ELSO between	Means	Hemorrhage DIC	Carotid VA: 96 (6%)<br>Femoral VA : 11 (10%)<br>VV: 35 (4%)	<0.05 car vs VV

			1993 and 2007				
Mode of cannulation (n (%))	2617 children with respiratory failure	Rollins, 2012	Retrospective ELSO between 1993 and 2007	Means	Clot in bladder	Carotid VA: 214 (14%)<br>Femoral VA : 19 (16%)<br>VV: 174 (18%)	Carotid arterial vs VV, P < .05.
Mode of cannulation (n (%))	2617 children with respiratory failure	Rollins, 2012	Retrospective ELSO between 1993 and 2007	Means	CNS bleed	Carotid VA: 98 (7%)<br>Femoral VA : 2 (2%)<br>VV: 41 (4%)	Carotid arterial vs VV, P < .05. Carotid vs femoral artery, P < .05.
Mode of cannulation, Carotid<br>Femoral<br><br>VV<br>(n (%))	2617 children with respiratory failure	Rollins, 2012	Retrospective ELSO between 1993 and 2007	Means	CNS injury	ICH 171 (73%) no ICH 1345 (56%)<br>ICH 8 (3%) no ICH 108 (5%)<br>ICH 54 (23%) no ICH 931 (39%)	<0.05<br><br>NS<br><br><0.05
Mode of cannulation percutaneous cannulation<br>Venous<br>Arterial (n (%))	2617 children with respiratory failure	Rollins, 2012	Retrospective ELSO between 1993 and 2007	Means	CNS injury	ICH 25 (11%) No ICH 454 (19%)<br>ICH 11 (5%) No ICH 90 (4%)	<0.05<br><br>NS
Mode of cannulation (n (%))	2617 children with respiratory failure	Rollins, 2012	Retrospective ELSO between 1993 and 2007	Means	CNS infarct	Carotid VA: 87 (6)<br>Femoral VA : 6 (5)<br>VV: 18 (2)	Carotid arterial vs VV, P < .05.
Mode of cannulation (n (%))	2617 children with respiratory failure	Rollins, 2012	Retrospective ELSO between 1993 and 2007	Means	Central nervous system bleed or infarct	Carotid VA: 171(11)<br>Femoral VA : 8 (7)<br>VV: 54 (6)	Carotid arterial vs VV, P < .05.
Mode of cannulation (VV/VA)	32 infants with PPHN who required ECMO	Doymaz, 2015	Retrospective chart review	Means	ICH	ICH VV 4/VA 7, no ICH VV 9/VA 12	0.722

Mode of cannulation VV ECMO	917 children with ICH from ELSO	Hardart, 1999	Retrospective ELSO review between 1992-1995	Multivariate	ICH	OR 0.97 (95% CI 0.73-1.30)	NS
Mode of cannulation VV ECMO (%)	274 patients with 299 ECMO runs. 38 circuit changes	Maul, 2020	Retrospective analysis	Means	Circuit change	Circuit change 6%, No circuit change 17%	NS
Pump type (centrifugal vs roller pump)	Propensity score matched analysis with 274 roller pump vs 274 centrifugal	Barret, 2013	Retrospective ELSO review from Jan 2007 to Dec 2009	Means	Mechanical complications	1.14 (IQR 0.76-1.71)	0.535
Pump type (centrifugal vs roller pump)	Propensity score matched analysis with 274 roller pump vs 274 centrifugal	Barret, 2013	Retrospective ELSO review from Jan 2007 to Dec 2009	Means	Circuit thrombosis	1.26 (IQR 0.73–2.18)	0.405
Pump type (centrifugal vs roller pump)	Propensity score matched analysis with 274 roller pump vs 274 centrifugal	Barret, 2013	Retrospective ELSO review from Jan 2007 to Dec 2009	Means	DIC - Bleeding complications	0.51 (IQR 0.23–1.17)	0.108
Pump type (centrifugal vs roller pump)	Propensity score matched analysis with 274 roller pump vs 274 centrifugal	Barret, 2013	Retrospective ELSO review from Jan 2007 to Dec 2009	Means	Cannula or surgical site bleeding	1.07 (IQR 0.75–1.53)	0.714
Pump type (centrifugal vs roller pump)	Propensity score matched analysis with 274 roller pump vs 274 centrifugal	Barret, 2013	Retrospective ELSO review from Jan 2007 to Dec 2009	Means	Hemolysis	4.03 (IQR 2.37–6.87)	<0.01
Pump type (centrifugal vs roller pump)	Propensity score matched analysis with 274 roller pump vs 274 centrifugal	Barret, 2013	Retrospective ELSO review from Jan 2007 to Dec 2009	Means	Cardiac tamponade	1.59 (IQR 0.67–3.73)	0.287

Pump type (centrifugal vs roller pump)	Propensity score matched analysis with 274 roller pump vs 274 centrifugal	Barret, 2013	Retrospective ELSO review from Jan 2007 to Dec 2009	Means	Pulmonary hemorrhage	0.54 (IQR 0.27–1.10)	0.085
Pump type (centrifugal vs roller pump)	Propensity score matched analysis with 274 roller pump vs 274 centrifugal	Barret, 2013	Retrospective ELSO review from Jan 2007 to Dec 2009	Means	GI hemorrhage	0.85 (IQR 0.28–2.57)	0.779
Pump type (centrifugal vs roller pump)	Propensity score matched analysis with 274 roller pump vs 274 centrifugal	Barret, 2013	Retrospective ELSO review from Jan 2007 to Dec 2009	Means	ICH	1.34 (IQR 0.79–2.28)	0.281
Pump type (centrifugal vs roller pump)	Propensity score matched analysis with 274 roller pump vs 274 centrifugal	Barret, 2013	Retrospective ELSO review from Jan 2007 to Dec 2009	Means	Cerebral infarct	1.17 (IQR 0.62–2.22)	0.62 in total, 45 without circuit change, 17 with circuit change6
ECMO flow (ml/kg/min)	36 cases (ICH & stroke) vs 36 controls (no ICH/stroke)	Anton-Martin, 2017	Retrospective single center individually matched case-control study	Means	ICH & stroke	Cases: 121 (IQR 53–223) Controls: 130.5 (IQR 53.5–207)	0.34
Pump flow at either 4th h or 24th h (>75th percentile, mL/kg/min)	All children supported with VA ECMO for cardiac support between Jan 2002 and April, 2013	Werho, 2015	Retrospective review of ELSO registry between Jan 2002 and April 2013	Multivariate	Stroke	OR 1.10 (95% CI 0.85-1.41)	0.47

Metabolic risk factors for bleeding or thrombosis in pediatric ECMO patients.

HCO_3 in last blood gas prior to ECMO initiation	113 children with ICH versus 859 without	Goodwin, 1995	Retrospective ELSO review between 1986 and 1988	Means	ICH	ICH 20.9 (SD 0.6), no ICH 23.2 (SD 0.2)	<0.01
HCO_3	104 children with ICH from ELSO	Hardart, 1999	Retrospective ELSO review between 1992-1995	Univariate	ICH	RR 1.9	<0.01
HCO_3 in pre-ECMO blood gas	1898 children with congenital heart disease	Polito, 2015	Retrospective ELSO review between 2005-2010	Means	Stroke/ICH	Stroke/ICH: 20 (IQR 13-28)<br>No stroke/ICH: 22 (IQR 14-29)	Not mentioned
Pre-ECMO HCO_3 therapy (n (%))	1898 children with congenital heart disease	Polito, 2015	Retrospective ELSO review between 2005-2010	Means	Stroke/ICH	Stroke/ICH: 91 (33%)<br>No stroke/ICH: 473 (29%)	0.16
HCO_3 therapy	185 patients born at <37 weeks with ICH vs 1194 without	Hardart, 2004	Retrospective ELSO review between 1992-2000	Multivariate	ICH	OR 1.7 (95% CI 1.2-2.41)	<0.01
Lactate	145 post-cardiotomy patients on ECMO in excessive and non-excessive bleeding group	Nardell, 2009	Retrospective review of ELSO between Jan 1998 and Jun 2007	Means	Bleeding complications	0-6h: NE 7.6 (SD 4.8) E 9.9 (SD 11.6)<br>7-12h: NE 5.5 (SD 4.2) E 6.4 (SD 4.6)<br>13-24h:<br>NE 4.4 (SD 5.1)<br>E 7.7 (SD 7.0)<br>25-48h:<br>NE 3.1 (SD 3.4)<br>E 2.4 (SD 0.4)<br>49h-5d:<br>NE 2.2 (SD 2.0)	0.1<br><br>0.4<br><br>0.06<br><br><br>0.7<br><br><br>0.2

						E 1.6 (SD 0.3)	
Initial lactate levels	Non-surgical neonates on VV or VA ECMO with ICH vs without ICH	Grayck, 1995	Retrospective chart review between Jan 91 and Feb 94	Means	ICH	ICH 10 (SD 1.7), no ICH 6.4 (SD 0.8)	0.05
Highest lactate	32 infants with PPHN who required ECMO	Doymaz, 2015	Retrospective chart review	Means	ICH	ICH 5.76 (SD 4.41), no ICH 3.75 (SD 3.91)	0.335
Lowest lactate	32 infants with PPHN who required ECMO	Doymaz, 2015	Retrospective chart review	Means	ICH	ICH 1.143 (SD 0.51), no ICH 1.04 (SD 0.59)	0.704
Maximum lactate	Non-surgical neonates on VV or VA ECMO with ICH vs without ICH	Grayck, 1995	Retrospective chart review between Jan 91 and Feb 94	Means	ICH	ICH 12.4 (SD 2.5), 7.9 (SD 0.8)	0.04
pH	25 newborns with ICH vs 40 control patients	De Mol, 2008	Retrospective matched case control study	Means	ICH	ICH 7.23 (SD 0.15), no ICH 7.33 (SD 0.15)	0.01
pH	113 children with ICH versus 859 without	Goodwin, 1995	Retrospective ELSO review between 1986 and 1988	Means	ICH	ICH 7.28 (SD 0.25), no ICH 7.41 (SD 0.06)	<0.01
pH	2617 children with respiratory failure	Rollins, 2012	Retrospective ELSO between 1993 and 2007	Means	CNS injury	ICH 7.23 (IQR 7.11-7.33)<br>No ICH 7.29 (IQR 7.18-7.39)	<0.05

pH<br><7<br>7-<7.2<br>7.2-<7.35	104 children with ICH from ELSO	Hardart, 1999	Retrospective ELSO review between 1992-1995	Univariate	ICH	RR 3.9<br>RR 2.6<br>RR 1.5	<0.01<br><0.01<br><0.01
pH<br>6.4-7.18<br>7.19-7.29<br><7.29	2617 children with respiratory failure	Rollins, 2012	Retrospective ELSO between 1993 and 2007	Multivariate	CNS injury	OR 2.1 (95% CI 1.5-2.8)<br>OR 1.5 (95% CI 1.1-2.1)<br>OR 1 ref group	Not mentioned
Pre-ECMO pH<br>≤7.15<br>7.15–7.36<br>≥7.36	1898 children with congenital heart disease	Polito, 2015	Retrospective ELSO review between 2005-2010	Multivariate	Stroke/ICH	OR 1.5 (95% CI 1.1–2.1)<br>OR 1.1 (95% CI 0.8–1.5)<br>OR 1.0 (reference)	0.02<br>0.5
pH <7<br>pH 7-7.2	104 children with ICH from ELSO	Hardart, 1999	Retrospective ELSO review between 1992-1995	Means	ICH	OR 2.5 (95% CI 1.6-3.9)<br>OR 1.8 (95% CI 1.3-2.5)	<0.01<br><0.01
pH <7.0<br>pH 7.0-<7.2	185 patients born at <37 weeks with ICH vs 1194 without	Hardart, 2004	Retrospective ELSO review between 1992-2000	Multivariate	ICH	OR 2.41 (95% CI 1.27-4.56)<br>OR 1.65 (95% CI 1.10-2.45)	<0.01<br><0.01
Highest pH	32 infants with PPHN who required ECMO	Doymaz, 2015	Retrospective chart review	Means	ICH	ICH 7.48 (SD 0.02), no ICH 7.49 (SD 0.06)	0.818
Lowest pH	32 infants with PPHN who required ECMO	Doymaz, 2015	Retrospective chart review	Means	ICH	ICH 7.22 (SD 0.12), no ICH 7.24 (SD 0.13)	0.701
pH<7.3 in worst arterial gas	25 newborns with ICH vs 40 control patients	De Mol, 2008	Retrospective matched case control study	Univariate	ICH	OR 4.5 (95% CI 1.4-14.0)	-

Acidosis (n (%))	752 neonates with gestational age between 29 and 31 weeks (n=243) versus neonates born after 34 weeks of gestation (n=509)	Church, 2017	Retrospective ELSO review between 1976 and 2008	Means	ICH	ICH 7 (18%), no ICH 33 (83%)	0.94
Acidosis (n (%))	752 neonates with gestational age between 29 and 31 weeks (n=243) versus neonates born after 34 weeks of gestation (n=509)	Church, 2017	Retrospective ELSO review between 1976 and 2008	Means	Cerebral infarction	ICH 4 (10%), no ICH 36 (90%)	0.20
