# Supplementary material for: Challenges in Maintaining the Hemostatic Balance in Children Undergoing Extracorporeal Membrane Oxygenation: A Systematic Literature Review
Source: Front Pediatr. 2020 Dec 16;8:612467. doi: 10.3389/fped.2020.612467 (PMC7772234; doi:10.3389/fped.2020.612467)
Supplement: Supplementary Material 3 — Newcastle Ottawa Scores. [file Table_3.pdf]

Supplemental material 3 Newcastle Ottawa Scores.

| | Selection of cohorts | | | | Comparability | Outcome | | | |
|---|---|---|---|---|---|---|---|---|---|
| Author, year | representativeness of the exposed cohort | selection of the non-exposed cohort | Ascertainment of exposure | demonstration that outcome of interest was not present at start of study | Comparability of cohorts on the basis of the design or analysis controlled for confounders | Assessment of outcome | Was follow-up long enough for outcomes to occur | Adequacy of follow-up of cohorts | **TOTAL (out of 7)** |
| Anton-Martin, 2017 | 1 | 0 | 1 | 0 | 0 | 1 | 1 | 0 | 4 |
| Anton-Martin, 2017 | 0 | 0 | 1 | 1 | 0 | 1 | 1 | 0 | 4 |
| Barrett, 2013 | 1 | 0 | 1 | 0 | 0 | 1 | 1 | 0 | 4 |
| Church, 2017 | 1 | 0 | 1 | 0 | 2 | 1 | 1 | 0 | 6 |
| Dalton, 2017 | 1 | 0 | 1 | 0 | 2 | 1 | 1 | 0 | 6 |
| De Mol, 2008 | 1 | 0 | 1 | 1 | 2 | 1 | 1 | 0 | 7 |
| Doymaz, 2015 | 0 | 0 | 1 | 1 | 0 | 1 | 1 | 0 | 4 |
| Goodwin, 1995 | 1 | 0 | 1 | 0 | 0 | 1 | 1 | 0 | 4 |
| Grayck, 1995 | 0 | 0 | 1 | 1 | 0 | 1 | 1 | 0 | 4 |
| Hardart, 1999 | 1 | 0 | 1 | 1 | 2 | 1 | 1 | 0 | 7 |
| Hardart, 2004 | 0 | 0 | 1 | 1 | 2 | 1 | 1 | 0 | 6 |
| Irby, 2014 | 1 | 0 | 1 | 1 | 0 | 1 | 1 | 0 | 5 |
| Maul, 2020 | 1 | 0 | 1 | 1 | 1 | 1 | 1 | 0 | 6 |
| Muensterer | 1 | 0 | 1 | 1 | 0 | 1 | 1 | 0 | 5 |
| Nardell, 2009 | 0 | 0 | 1 | 0 | 1 | 1 | 1 | 0 | 4 |
| Polito, 2015 | 0 | 0 | 1 | 0 | 0 | 1 | 1 | 0 | 3 |
| Rollins, 2012 | 0 | 0 | 1 | 0 | 2 | 1 | 1 | 0 | 5 |
| Rozmiarek, 2004 | 1 | 0 | 1 | 0 | 0 | 1 | 1 | 0 | 4 |

| | | | | | | | | | |
|---|---|---|---|---|---|---|---|---|---|
| Saini, 2016 | 1 | 0 | 1 | 0 | 0 | 1 | 1 | 0 | 4 |
| Sell, 1986 | 1 | 0 | 1 | 1 | 0 | 1 | 1 | 0 | 5 |
| Stallion, 1994 | 1 | 0 | 1 | 1 | 0 | 1 | 1 | 0 | 5 |
| Werho, 2015 | 0 | 0 | 1 | 0 | 2 | 1 | 1 | 0 | 5 |
| Zahraa, 2000 | 0 | 0 | 1 | 0 | 0 | 1 | 1 | 0 | 3 |
